# From digital product diffusion to sustainable population health: a two-layer complex-network evolutionary game analysis of digital manufacturing and health-oriented sport consumption

**DOI:** 10.3389/fpubh.2026.1922940

**Published:** 2026-08-21

**Authors:** Shixu Chen, Junbao Lian, Weihan Hu, Tao Wang, Yang Cao

**Affiliations:** School of Physical Education and Sport Science, Fujian Normal University, Fuzhou, China

**Keywords:** complex-network evolutionary game, digital health manufacturing, health-oriented innovation diffusion, sport and health consumption, sustainable population health

## Abstract

The digital transformation of the sports goods manufacturing industry has become an important pathway for improving the supply capacity of sport and health products and stimulating health-oriented consumption demand. However, existing studies have paid limited attention to the coupled diffusion mechanism between supply-side technological upgrading and demand-side consumer adoption in networked contexts. To address this gap, this study develops a two-stage evolutionary game model on complex networks to analyze the co-evolutionary process between digital innovation technology adoption by sports goods manufacturing firms and consumer adoption of intelligent sport and health products. In the first stage, firms decide whether to adopt digital innovation technologies and engage in collaborative innovation. In the second stage, consumers decide whether to adopt digital sport and health products based on perceived product value and social influence within the consumption network. The results show that: (1) Policy incentives can effectively promote technological transformation among sports goods manufacturing firms and activate the sport and health consumption market. However, government subsidies on the firm side exhibit diminishing marginal effects, suggesting that a reasonable subsidy threshold should be established. (2) High-quality digital sport and health products are the core factor driving sport and health consumption. Improvements in firms’ digital capabilities and technology maturity can enhance product health utility, user experience, and market attractiveness. (3) When residents develop strong value recognition and willingness to use digital sport and health products, a more stable mutually reinforcing mechanism can be formed between firms’ digital technology adoption and consumer-side diffusion of health-oriented products. Conversely, under low-preference conditions, the supply side and the demand side may fall into a low-level diffusion state. By integrating complex-network evolutionary game analysis with innovation diffusion theory, this study reveals the dynamic collaborative diffusion mechanism of digital sport and health products. The findings extend the analytical framework for studying digital upgrading in sports manufacturing and provide theoretical and practical implications for promoting intelligent transformation and sustainable sport and health consumption.

## 1 Introduction

The deep integration of digital technologies with the sports goods manufacturing industry is reshaping the production and consumption of sport-and-health products (1).

With the rapid development of digital technology-enabled services, sports goods are no longer confined to conventional exercise equipment; instead, they are increasingly embedded in everyday sport, health, and lifestyle scenarios (2). In this context, digital sport-and-health products not only extend the functional boundaries of sports goods but also strengthen the reciprocal linkage between sports manufacturing and population health promotion (3). Accordingly, the digital upgrading of the sports goods manufacturing industry is not merely a matter of consumer-market expansion; it also represents an important pathway for increasing the supply of sport-and-health services and improving access to population health resources.

The diffusion of sport-and-health consumption should therefore not be understood as a one-way process of market growth. Rather, it is a co-evolutionary process in which firms’ technological upgrading and residents’ product adoption interact, generate feedback, and evolve dynamically over time (4). At its core, this process concerns whether digital health resources can be accessed, understood, trusted, and ultimately translated into everyday health-related actions by different groups of residents. Existing studies have examined industrial upgrading, digital transformation, innovation diffusion, and consumer health behavior from multiple perspectives (5, 6). However, existing studies have largely treated firm innovation adoption and consumer product adoption as separate processes, with limited attention to the coevolution of supply side upgrading and demand side health consumption. From an innovation diffusion perspective, firms adopt digital technologies to provide innovative products and services, while consumers evaluate and adopt them through individual decision making and social interaction. Consumer demand and adoption feedback, in turn, shape firms’ expectations of innovation returns and subsequent innovation decisions. Thus, diffusion is not a unidirectional process from technology supply to market adoption, but a dynamic interaction between supply and demand. How such interactions enable digital sport and health products to evolve from conventional consumer products into health support resources embedded in everyday life remains insufficiently understood.

Against this background, this study develops a two-stage evolutionary game model on complex networks to analyze the dynamic transformation mechanism through which digital sport-and-health products move from technological supply by firms to health-oriented adoption by residents. The first stage focuses on technology adoption among sports goods manufacturing firms, while the second stage examines consumers’ adoption of sport-and-health products. By coupling the innovation process on the firm side with the adoption process on the consumer side, this study constructs a dynamic complex-network framework characterized by two-way feedback between supply and demand. This framework is used to examine how policy incentives, technological maturity, data analytics capability, and consumer preferences jointly shape the diffusion of digital sport-and-health products, and to further reveal the internal mechanism by which such products may evolve into resources supporting population health. In doing so, this study offers theoretical and practical insights for improving the accessibility and equity of public health services.

## 2 Literature review and evolutionary mechanism

### 2.1 Literature review

As an important component of the broader sports industry system, the sports goods manufacturing industry influences not only the quality of supply in the sports consumption market but also the ways in which residents participate in physical activity and develop healthy lifestyle habits.

During the traditional stage of industrial development, the sports goods manufacturing industry was primarily centered on physical production, and firms’ competitive advantages were mainly derived from expanded production scale, manufacturing techniques, and sales channels. With the development of digital manufacturing technologies, however, the role of this industry is no longer limited to improving production efficiency. Sports goods manufacturers are gradually shifting from product-oriented manufacturing toward digitally enabled product–service provision (7). Smart sensing devices, wearable technologies, and sport data analytics are increasingly being embedded into sports equipment, enabling some sports goods to evolve beyond simple exercise tools into digital sport-and-health products with functions related to health management and behavioral intervention (1, 8–10). Existing studies have begun to examine the effects of digital transformation and servitization among sports goods manufacturing firms. For example, Lu et al., using data from Chinese listed sports goods manufacturing firms, found that servitization in this sector remains at an exploratory stage; an increase in servitization level does not necessarily improve firm performance and may even lead to a servitization paradox. Hu et al., (9) drawing on the TOE framework and fsQCA method, further showed that the servitization upgrading of sports goods manufacturing firms is not directly determined by a single factor, such as digital innovation capability or government support, but rather emerges from multiple configurational pathways formed by technological, organizational, and environmental conditions. These studies provide important evidence for understanding the digital and service-oriented transformation of the sports goods manufacturing industry. However, their analytical focus remains largely at the firm-internal level, with insufficient attention paid to the dynamic coupling between firm innovation behavior and consumer adoption behavior.

From the perspective of industrial innovation, the digital upgrading of the sports goods manufacturing industry should not be viewed as a purely internal process of technological optimization within individual firms, but rather as a dynamic process embedded in a broader industrial ecosystem. Research on digital innovation suggests that innovation actors in digital contexts are increasingly distributed, and that innovation processes depend more heavily on the joint participation of platforms, users, partners, and external knowledge resources (4, 11). Collaborative innovation can influence new product performance through product innovation capability and process innovation capability, but such effects often depend on firms’ ability to absorb external knowledge and integrate resources (12). Therefore, whether sports goods manufacturing firms choose to engage in digital innovation depends not only on their own cost–benefit considerations but also on industrial ecosystem relationships, such as neighboring firms and cooperative structures.

From the perspectives of public health and physical activity promotion, the value of digital sport-and-health products also lies in their potential to promote residents’ participation in physical activity. High-quality reviews have shown that wearable activity trackers generally help increase physical activity levels and improve certain health-related indicators in some populations (13, 14). Systematic reviews focusing on children and adolescents have also suggested that wearable activity trackers may increase daily step counts, although their effects on moderate-to-vigorous physical activity remain inconsistent (15). These findings indicate that digital sports goods have potential health-promoting value. However, their actual diffusion does not depend solely on the technology itself; it is also shaped by consumers’ user experience, trust in data, and sustained use behavior (16, 17). Therefore, it is insufficient to interpret digital sport-and-health products merely as outcomes of firms’ technological upgrading. Their diffusion mechanism needs to be understood through the interaction between supply-side technology adoption and demand-side consumer adoption. In this sense, the transformation of the sports goods manufacturing industry is no longer simply a technological upgrading of production methods, but a joint reconstruction of product forms, service models, and health-related value functions.

Innovation diffusion theory provides a valuable perspective for understanding how new technologies and practices spread within social systems. The theory identifies five key attributes influencing innovation adoption, including relative advantage, compatibility, complexity, trialability, and observability. Relative advantage refers to the perceived benefits of an innovation over existing alternatives. Compatibility reflects its consistency with adopters’ values, needs, and experiences. Complexity refers to the perceived difficulty of understanding or using the innovation. Trialability indicates the extent to which it can be tested before full adoption, while observability reflects the visibility of its outcomes and benefits to others. Together, these attributes help explain why similar innovations may exhibit different diffusion trajectories across social systems (18). In the fields of public services and digital health, innovation diffusion is further shaped by users’ needs, communication channels, digital capabilities, and institutional support. Consequently, it is generally regarded as a dynamic process involving both individual decision-making and social interaction (19, 20). Within the context of digital public health, the diffusion of innovative products depends not only on sports goods manufacturing firms’ digital manufacturing capabilities and service extension capacity, which enable products to integrate digital health functions such as health monitoring and exercise feedback, but also on consumers’ willingness to adopt these products. Consumer adoption is influenced not only by the technological availability of digital sport and health products, but also by perceived health benefits, affordability, privacy trust, and evaluations from other users (21, 22).

Therefore, the diffusion of digital sport and health products should not be viewed simply as a process of market penetration, but rather as a dynamic process in which consumers gradually progress from evaluating technological innovations to adopting them. Building upon this perspective, evolutionary game theory and complex network theory provide an appropriate analytical framework for modeling the dynamic diffusion of digital sport and health products. Unlike classical game theory, which assumes fully rational actors seeking static equilibrium, evolutionary game theory emphasizes gradual strategy adjustment through bounded rationality and imitation, making it particularly suitable for analyzing strategic interactions between firms and consumers under uncertain market conditions. Complex network theory further captures the relational structures and heterogeneous interactions underlying innovation diffusion, such as the leading role of core firms within enterprise networks and clustering effects in consumer communication networks (23–25). By integrating evolutionary game theory with complex network analysis, this study links micro-level strategic adaptation with macro-level diffusion outcomes, thereby revealing the dynamic coupling mechanism between supply-side digital technological upgrading and demand-side health-oriented consumption adoption in support of sustainable population health coverage.

In summary, this study places sports goods manufacturing firms and consumers within a unified dynamic evolutionary framework. It develops a two-stage complex-network evolutionary game model that couples firm innovation networks with consumer adoption networks, aiming to reveal how policy support, firms’ digital capabilities, collaborative innovation relationships, and consumer preferences jointly influence the diffusion of digital sport-and-health products.

### 2.2 Evolutionary mechanism

**Figure 1:**
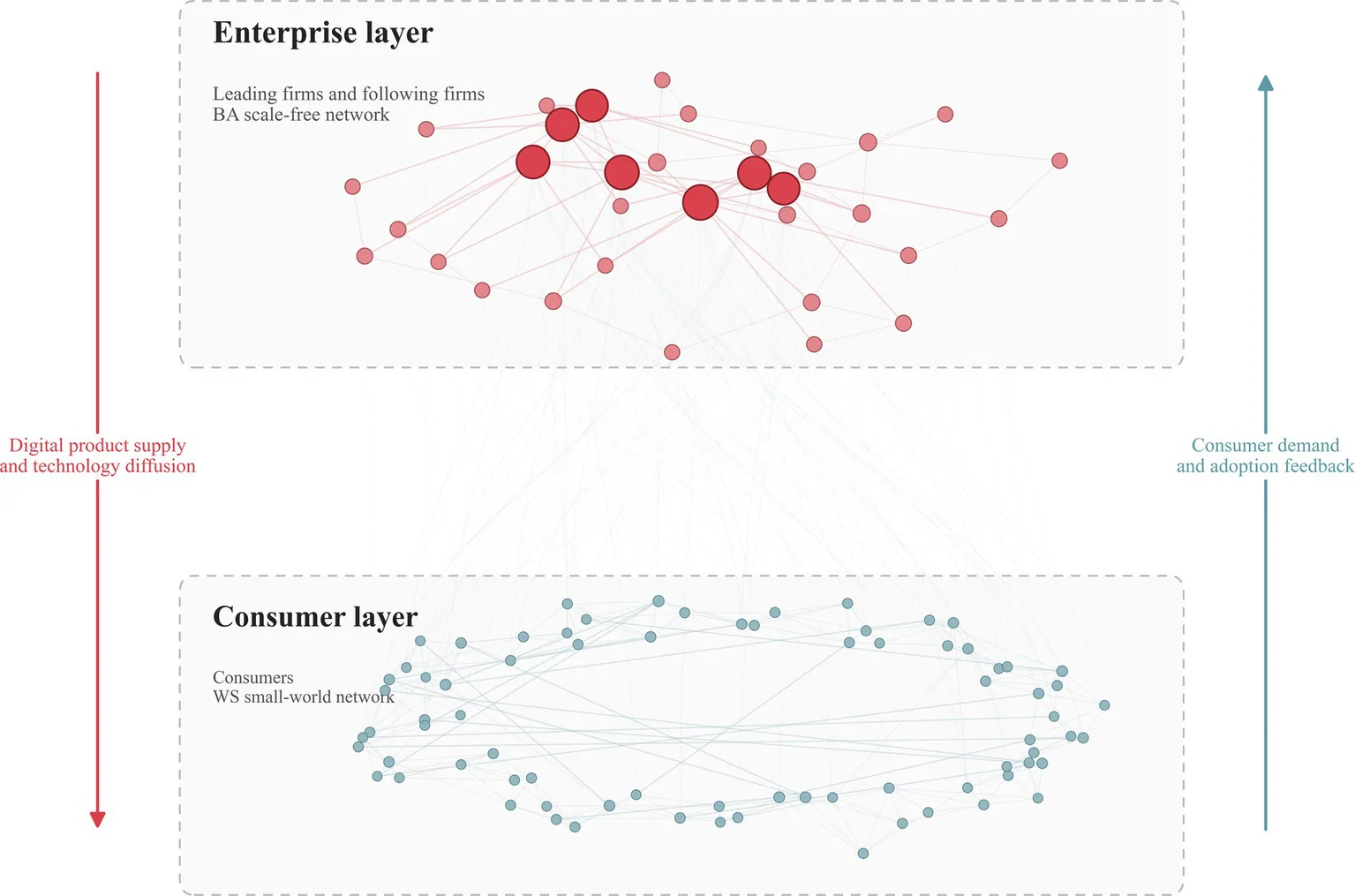
Framework of the two layer complex network evolutionary game model. PS: The enterprise layer consists of leading and following firms and is modeled using a scale-free network to characterize innovation diffusion among firms. The consumer layer employs a small-world network to capture information diffusion and product adoption among consumers. Firms influence consumer adoption through the supply of digital sport and health products, while consumer demand feedback, in turn, affects firms’ digital innovation decisions. The two network layers thus form a co-evolutionary process through a bidirectional feedback mechanism.

Driven by the practical need for sustainable population health, the diffusion of digital sport and health products is not only a market outcome of the digital transformation of the sports goods manufacturing industry but also a key process through which sport and health resources are continuously supplied and translated into residents’ health behaviors. Accordingly, this diffusion process involves both supply-side digital technological upgrading by sports goods manufacturing firms and demand-side evolution in consumer health needs, value perception, and product adoption. Based on this perspective, this study examines the evolutionary mechanisms of digital sport and health product diffusion from the perspectives of supply-side digital technological upgrading and demand-side health-oriented consumption adoption, providing the theoretical foundation for the subsequent two-stage evolutionary game model on complex networks. The conceptual framework is presented in Figure 1.

#### 2.2.1 Sports goods manufacturing industry

Sports goods manufacturing firms are the primary suppliers of digital sport-and-health products. In response to the development of digital technologies and the upgrading of sport-and-health consumption, firms may either adopt digital innovation technologies or maintain traditional production modes.

When firms choose to adopt digital innovation technologies, they can improve production efficiency and expand the service-extension capacity of their products. This enables sports goods to incorporate functions such as physical activity monitoring, health feedback, personalized guidance, and data-based services (26). As a result, firms may develop differentiated advantages in market competition and enhance the attractiveness of their products to consumers (27). However, digital innovation is often accompanied by high research and development investment and technological risks (28). In particular, when firms lack accurate expectations regarding innovation outcomes or when consumer demand remains insufficient, they may be more inclined to maintain existing traditional production modes to ensure operational stability under risk-averse considerations.

#### 2.2.2 Consumers

Consumers are the demand-side actors in the diffusion of digital sport-and-health products. When faced with such products, consumers generally have two strategic choices: active adoption or rational wait-and-see.

Consumer adoption of digital sport-and-health products is not a simple one-time purchase decision. Rather, it is a rational judgment formed after consumers comprehensively evaluate product value, health utility, ease of use, and perceived risk costs (29). When digital sport-and-health products provide higher health utility, stronger functional reliability, and better user experience, consumers are more likely to regard them as effective health management tools and gradually shift toward an active adoption strategy (30). Conversely, when consumers perceive insufficient product value or high usage-related risks and costs, their willingness to adopt may be constrained, leading them to maintain a wait-and-see attitude (31).

At the same time, consumer adoption behavior also exhibits characteristics of social diffusion. The actual experiences of early adopters are often transmitted through demonstration effects and word-of-mouth evaluations. When positive experiences spread within a consumer group, the proportion of adopters may increase in a self-reinforcing manner. By contrast, the diffusion of negative information may weaken subsequent adoption intentions (32, 33). Therefore, the strategic evolution of consumer groups is a dynamic process jointly shaped by individual rational choice and group-level interactive diffusion.

#### 2.2.3 Interaction mechanism between firms and consumers

The diffusion of digital sport and health products depends not only on the strategic evolution of firms and consumers but also on the continuous interaction between the supply and demand sides. Consumer demand serves as an important market signal shaping firms’ expectations of innovation returns and perceived risks. As consumers become more willing to adopt digital sport and health products, firms anticipate greater market potential and are more likely to invest in digital innovation. Positive word-of-mouth within consumer networks further reduces market uncertainty, while continuous feedback on product functionality and health benefits enables firms to optimize products and improve innovation efficiency. Therefore, consumer adoption is not only an outcome of firms’ innovation but also an important feedback mechanism that stimulates continuous digital innovation.

Conversely, firms’ digital innovation decisions also shape consumer adoption. Digital technologies can enhance product functionality, health services, and user experience, thereby strengthening consumers’ perceived value and trust in digital sport and health products. However, if digital upgrading results in higher prices, greater usage complexity, or concerns about data privacy, consumer adoption may be discouraged until technology maturity and trust are sufficiently improved.

## 3 Materials and methods

### 3.1 Model description

The model is operationalized based on the core dimensions of Innovation Diffusion Theory. The five perceived attributes of innovation have distinct but interrelated manifestations at the firm and consumer levels. Relative advantage encompasses the expected economic and competitive benefits of digital innovation for firms and the additional health and service value perceived by consumers, such as health feedback and personalized guidance. Compatibility reflects the alignment of digital innovation with firms’ existing resources and production practices, as well as its fit with consumers’ health needs and value perceptions. Complexity arises from the innovation risks and costs faced by firms and the difficulties perceived by consumers in product use, including affordability and privacy concerns. Trialability reflects opportunities for firms to experiment with digital technologies on a limited scale and for consumers to experience products before full adoption. Policy incentives and consumer subsidies may facilitate initial trials by reducing costs and uncertainty. Observability operates through network demonstration and social learning. Firms learn from the innovation outcomes of other firms, while consumers adjust their adoption decisions based on other users’ experiences and evaluations within social networks. The theoretical dimensions and corresponding model mechanisms are presented in Tables 1, 2.

**Table 1:** Parameters and definitions.

Symbol	Definition
Decision variables	
x	The proportion of firms adopting digital innovation technologies
1−*x*	The proportion of sports goods manufacturing firms maintaining traditional production modes
φ (*x*)	The proportion of leading firms adopting digital technologies
ϕ (*x*)	The proportion of following sports goods manufacturing firms adopting digital innovation technologies
*y*	The corresponding proportion of consumers purchasing digital sport and health products
1−*y*	The proportion of consumers purchasing traditional products
Parameters	
*λ*	Preference of sports goods manufacturing firms for digital innovation technologies, intelligent manufacturing, and data-driven operations
*α*	Consumers’ perceived value of the convenience, health feedback, and intelligent user experience of digital sport and health products
S_1_	Government subsidy for digital innovation provided to leading sports goods manufacturing firms
S_2_	Government subsidy for digital innovation provided to following sports goods manufacturing firms
S_3_	Consumer voucher subsidy or purchase incentive provided by the government for digital sport and health products
C_d_	Cost incurred by firms when adopting digital innovation technologies
C_t_	Cost incurred by firms when maintaining traditional sports goods production modes
*τ*	Technology maturity of digital sport and health products, where *τ*∈[0,1]
$c_{d}e^{-\tau }$	Digital technology cost that decreases with increasing technology maturity; the more mature the technology, the lower the costs of digital production and research and development for firms
*ŋ*_L_	Data analytics capability and innovation capability of leading sports goods manufacturing firms
*ŋ*_F_	Data analytics capability and innovation capability of following sports goods manufacturing firms
*ŋ*_L_/*τ*^2^	Risk coefficient faced by leading firms during digital technology learning and product innovation
*ŋ*_F_/*τ*^2^	Risk coefficient faced by following firms during digital technology learning and product innovation
n	Cost incurred by firms in managing the research and development, application, and market uncertainty of digital sport and health products
m	Cost incurred by firms in collaborative innovation with universities, research institutions, and digital technology enterprises
${\mathrm{me}}^{-x}$	Collaboration cost that decreases as the proportion of digital technology firms increases; the higher the proportion of digitalized firms, the lower the costs of learning, communication, and collaborative innovation
F	Market competition pressure faced by firms that continue to maintain traditional technologies when other firms adopt digital innovation technologies, resulting in declining market share and weakened product competitiveness
R	Basic satisfaction utility obtained by consumers when purchasing traditional sport products
Δw	Incremental value generated by digital sport and health products, including health monitoring, exercise feedback, and personalized services
*ατ*Δw	Additional sport and health utility obtained by consumers due to stronger digital health preferences and improved technology maturity
P_d_	Price of digital sport and health products
P_t_	Price of traditional sport products
$p_{d}e^{-\tau }$	Price of digital products that decreases with increasing technology maturity; the more mature the technology, the lower the effective price of digital sport and health products
N	Number of consumers in the sport and health consumption market
qd	Market demand for digital sport and health products
q_t_	Market demand for traditional sport products

**Table 2:** Dimensions of innovation diffusion theory and their operationalization in the proposed model.

Innovation diffusion	Theory dimension	Theoretical meaning	Corresponding model parameters	Specific mechanism in the model
Relative advantage	The perceived superiority of an innovation over existing alternatives.	*λ*, S₁, S₂, Cd/Ct, *τ*, F, qd/qt *α*, S₃, Δw, *ατ*Δw, R, Pd/Pt	For firms, it reflects the cost and competitive advantages of digital technologies over traditional technologies; for consumers, it reflects perceived advantages in health functions, service value, and price. Demand feedback dynamically affects firms’ expected advantages of digital technology adoption.	Relative advantage determines whether products can become reliable, affordable, and attractive health resources.
Compatibility	The consistency of an innovation with adopters’ values, needs, and experiences.	*α*, R, *ατ*Δw *η*L/*η*F	Consumer preference reflects alignment with health needs and exercise habits, while basic health value and digital capabilities represent consumer needs and firms’ organizational compatibility, respectively.	Compatibility determines whether product adoption can be translated into health-related behavior.
Complexity	The perceived difficulty of understanding or using an innovation.	Cd, *τ*, *η*L/*η*F, n, n*τ*^2^/*η*L, n*τ*^2^/*η*F, m, m·e^(−x)^	Technological maturity and digital capabilities reduce uncertainty, implementation difficulty, and risk costs, while greater firm adoption reduces collaborative complexity through knowledge sharing.	Excessive complexity may constrain product supply and reduce access to health resources.
Trialability	The extent to which an innovation can be tested before full adoption.	S₁, S₂, S₃, *τ*	Subsidies and cost reductions lower the uncertainty and economic barriers to initial adoption.	Trialability improves initial product accessibility by lowering first-use barriers.
Observability	The extent to which an innovation’s outcomes and benefits are observable to others.	x, *φ*(x), *ϕ*(x), F, m·e^(−x)^, BA: NE, di, dj; y, WS: M, d, p	Firms observe others’ adoption strategies and market outcomes, while consumers assess product effects through word of mouth and peer experiences. Network structures shape the pathways and speed of information diffusion.	Observability shapes the diffusion of product experiences and health information across population groups.

Policy and network factors indirectly influence innovation attributes and adoption through costs and information diffusion.

*H1*. Strategy sets and evolutionary updating of firms and consumers.

Sports goods manufacturing firms make strategic choices according to market demand and their own capabilities. They have two pure strategies. The first is to adopt digital innovation technologies and actively engage in collaborative innovation with universities and research institutions. The second is to maintain traditional technologies and existing production modes. The two strategies are denoted as digital innovation and traditional technology, respectively. Let (*x*) and (1−*x*) represent the proportions of firms choosing digital innovation and traditional technology, where x∈[0,1]. Among them, the proportion of leading firms choosing active collaborative innovation is denoted by φ (*x*), while the proportion of following firms choosing active collaborative innovation is denoted by ϕ (*x*). Consumers also have two pure strategies when facing upgraded sport and health products. They may actively purchase and use sport and health products, or they may adopt a rational wait and see strategy and maintain their existing consumption habits, such as continuing to use traditional fitness equipment. These two strategies are denoted as active purchase and rational wait and see, respectively. Let (*y*) and (1−*y*) represent the proportions of consumers choosing active purchase and rational wait and see, where y∈[0,1]. During the evolutionary process, the proportion of firms choosing collaborative digital innovation and the proportion of consumers choosing active health consumption change continuously over time. Therefore, (*x*) and (*y*) are functions of time (*t*). Firms and consumers compare payoffs and update their strategies through nodes embedded in scale free networks and small world networks. Exploring the micro level evolutionary process of individual nodes helps reveal the macro level diffusion patterns of digital sport and health products.

*H2*. Digital innovation capability, technology maturity, and policy incentives of firms.

Let (*λ*) denote firms’ preference for digital transformation. The costs incurred by sports goods manufacturing firms under digital innovation and traditional technology production modes are denoted as (*C_d_*) and (*C_t_*), respectively, where (*C_d_* > *C_t_*). With continuous innovation and deeper collaboration with universities and research institutions, firms can improve the technology maturity level of digital sport and health products, denoted as (*τ*), where τ∈[0,1] . As the level of production technology improves, production costs gradually decline, while product performance and expected returns are enhanced. The data analytics capabilities of leading firms and following firms are denoted as (*ŋ*_L_) and (*ŋ*_F_), respectively. Differences in these capabilities lead firms to face different risks in the processes of technology learning and product innovation. The capability adjusted risk cost coefficients of leading firms and following firms are represented by (*ŋ*_L_/*τ*^2^) and ( ŋF/τ2 ), respectively, and (*n*) denotes the intensity of risk and loss costs. Firms may reduce innovation risks through cooperation and knowledge exchange with other firms adopting digital innovation technologies as well as with universities and research institutions, and the cooperation cost is denoted as (*m*). As the proportion of firms choosing collaborative digital innovation increases, the risks and loss costs associated with research and development decline in a nonlinear manner, which can be represented by ( ${\mathrm{me}}^{-x}$ ). Under policy guidance aimed at promoting the upgrading of sports goods and the high quality development of sport and health services, the government provides policy incentives to sports goods manufacturing firms. Firms choosing digital innovation technologies receive additional government rewards or subsidies, denoted as (S_1_) and (S_2_). In contrast, firms that maintain traditional technologies while other firms adopt digital innovation will face increasing market competition pressure, denoted as (*F*).

*H3*. Consumer preference, health utility, and demand side incentives.

In the market, consumers’ strategic choices are mainly influenced by consumer preference, denoted as (*α*). Consumer preference reflects consumers’ health concepts, their value perception of technological features and user experience, and their overall judgment of sport and health products when making purchase decisions. Assume that there are (*N*) consumers in the market. The demand for digital sport and health products is denoted as (*q_d_*), while the demand for traditional products is denoted as (*q_t_*). The price of new sport and health products is denoted as (*P*). As production technology gradually matures, the market price of digital sport and health products is adjusted accordingly and can be expressed as ( $p_{d}e^{-\tau }$ ). Consumers obtain a basic level of demand satisfaction, denoted as (*R*), when purchasing products that meet their needs. Each sport and health product releases a certain health effect, denoted as (Δ*w*). This health effect is influenced by the level of production technology or technology maturity (*τ*). For example, higher technology maturity may improve the accuracy of health monitoring, enhance comfort, and strengthen personalized feedback. Therefore, consumers who purchase sport and health products obtain additional health utility represented by (*τ*Δ*w*), whereas consumers who do not purchase these products do not obtain this additional health utility. When the government stimulates health consumption from the demand side, it provides consumer subsidies in the form of vouchers. The subsidy received by consumers when purchasing sport and health products is denoted as (*S*_3_).

### 3.2 Evolutionary model

#### 3.2.1 Network evolutionary dynamics model

Firms tend to establish cooperative relationships with, or compare their strategies against, firms with greater influence in the industry. This behavioral tendency is consistent with the topological characteristics of a scale-free network, in which a small number of key nodes occupy a large proportion of network connections, whereas most nodes maintain relatively low degrees. Accordingly, the degree distribution of nodes in the network follows a power-law pattern (Equation 1-3). The scale-free network is initialized with (*N*) isolated nodes, where (*N*) is a positive integer. At each time step (*t*), a newly added node connects to an existing node (*i*) in the network according to a preferential attachment mechanism, and repeated connections are not allowed. The probability that the new node connects to node (*i*) is defined as follows:


$$p(i)=\frac{d_{i}(t)}{\sum_{j=1}^{N_{n}}d_{j}(t)}$$ (1)

In the complex network, firm (*V_i_*) randomly selects a directly connected firm (*V_j_*) as its network neighbor. Here, ( $d_{i}(t)$ ) and ( $d_{j}(t)$ ) denote the degrees of firms (*V_i_*) and (*V_j_*) in the network at time (*t*), respectively, while $\sum_{j=1}^{N_{n}}d_{j}(t)$ represents the total degree of all nodes in the network at time. This setting reflects the preferential attachment process through which influential firms are more likely to attract new connections, thereby generating the heterogeneous connectivity structure of the enterprise innovation network.


$$p(i\to j)=\frac{1}{1+\exp[(U_{j}-U_{i})/k]}$$ (2)

In the calculation, ( $U_{i}$ ) and ( $U_{j}$ ) denote the payoffs obtained by firms (*V_i_*) and (*V_j_*) when they adopt strategies (*s_i_*) and (*s_j_*), respectively. The parameter (*k*) is the white noise coefficient, which measures the intensity of the influence of external factors on individual behavioral decisions.


$$\begin{array}{c} p(i\to j)=\sum_{j\in \partial_{c}}U_{j}^{\lambda }/U_{i}^{\lambda } \end{array}$$ (3)

The small-world network is constructed as a nearest-neighbor coupled network consisting of (M) nodes. In the initial network, each node (i) is connected to (d/2) nearest neighbors on both its left and right sides, where (d) is an even number and (1 < d/2 < M). Subsequently, after the initial connections are formed, consumer (i) in the market may rewire its connection to another consumer (j) with probability (p(i → j)). Here, (*λ*) represents consumers’ preference tendency toward different sport and health products or services.

#### 3.2.2 Two-stage evolutionary game model

(1) Enterprise innovation stage.

In the enterprise innovation stage, firms may obtain different payoffs when choosing collaborative digital innovation technologies. For pillar leading firms, the expected payoff from adopting collaborative digital innovation technologies is denoted as ( $\pi_{L}^{C}$ ). For competitive peripheral firms, the expected payoff from adopting collaborative digital innovation technologies is denoted as ( $\pi_{L}^{T}$ ). Since traditional technologies are relatively mature in the market, firms that choose traditional production technologies obtain relatively stable expected payoffs, regardless of their development level. The expected payoffs of firms adopting traditional technologies are denoted as ( $\pi_{F}^{C}$ ) and ( $\pi_{F}^{T}$ ), respectively. Based on this setting, this study constructs a payoff matrix for pillar leading firms and competitive peripheral firms under different production strategies, as shown in Table 3.

**Table 3:** Payoff matrix of leading firms and following firms.

Leading firms and following firms		Leading firms	
		Digital innovation technology ( $\pi_{L}^{C}$ )	Traditional technology ( $\pi_{L}^{T}$ )
Following firms	Digital innovation technology ( $\pi_{F}^{C}$ )	$(p_{d}e^{-\tau }-c_{d}e^{-\tau })q_{d}+\lambda q_{d}+S_{1}-\frac{n\tau^{2}}{\eta_{L}}-me^{-x}+R+\text{ατΔw}$ $(p_{t}-c_{t})q_{t}-F+R$	$(p_{d}-c_{d})q_{d}-F+R$ , $(p_{d}e^{-\tau }-c_{d}e^{-\tau })q_{d}+\lambda q_{d}+S_{2}-\frac{n\tau^{2}}{\eta_{F}}-me^{-x}+R+\text{ατΔw}$
	Traditional technology ( $\pi_{F}^{T}$ )	$(p_{d}e^{-\tau }-c_{d}e^{-\tau })q_{d}+\lambda q_{d}+S_{1}-\frac{n\tau^{2}}{\eta_{L}}+R+\text{ατΔw}$ $(p_{t}-c_{t})q_{t}-F+R$	$(p_{d}-c_{d})q_{d}-F+R$ $(p_{t}-c_{t})q_{t}-F+R$

The expected payoff of firms can be expressed as:

The expected payoff of leading firms choosing digital innovation technologies is given by:


$$\begin{array}{c} \pi_{L}^{C}=(p_{d}e^{-\tau }-c_{d}e^{-\tau })q_{d}+\lambda q_{d}+S_{1}-\frac{n\tau^{2}}{\eta_{L}}-me^{-x}+R+\alpha \tau \Delta w \end{array}$$ (4)

The expected payoff of leading firms maintaining traditional production modes can be expressed as:


$$\begin{array}{c} \pi_{L}^{T}=(p_{t}-c_{t})q_{t}-F+R \end{array}$$ (5)

The expected payoff of following firms adopting digital innovation technologies can be expressed as:


$$\begin{array}{c} \pi_{F}^{C}=(p_{d}e^{-\tau }-c_{d}e^{-\tau })q_{d}+\lambda q_{d}+S_{2}-\frac{n\tau^{2}}{\eta_{F}}-me^{-x}+R+\alpha \tau \Delta w \end{array}$$ (6)

The expected payoff of following firms maintaining traditional production modes can be expressed as:


$$\begin{array}{c} \pi_{F}^{T}=(p_{t}-c_{t})q_{t}-F+R \end{array}$$ (7)

Based on the payoff matrix, the replicator dynamic equations of leading firms and following firms can be derived:


$$\begin{array}{l} F(x)=\frac{\mathrm{d\psi}}{\mathrm{dt}}=\psi (1-\psi )\cdot [\begin{array}{l} \phi (x)(\text{πHCC}-\text{πHNC})\\ +(1-\phi (x))(\text{πHCN}-\mathrm{\pi NN}) \end{array}]\\ =\psi (1-\psi )[\begin{array}{l} (p_{d}e^{-\tau }-c_{d}e^{-\tau })q_{d}+\lambda q_{d}\\ +S_{1}+R+\text{ατΔw}-\frac{n\tau^{2}}{\eta_{L}}\\ -{\mathrm{me}}^{-x}-(p_{t}-c_{t})q_{t}+F \end{array}] \end{array}$$ (8)

$$\begin{array}{l} F(y)=\frac{d\varphi }{\mathrm{dt}}=\varphi (1-\varphi )\;[\begin{array}{l} \varphi (x)(\pi \mathrm{LCC}-\pi \mathrm{LCN})\\ +(1-\varphi (x))(\text{πLNC}-\mathrm{\pi NN}) \end{array}]\\ =\varphi (1-\varphi )[\begin{array}{l} (p_{d}e^{-\tau }-c_{d}e^{-\tau })q_{d}+\lambda q_{d}\\ +S_{2}+R+\text{ατΔw}-\frac{n\tau^{2}}{\eta_{F}}\\ -me^{-x}-(p_{t}-c_{t})q_{t}+F \end{array}] \end{array}$$ (9)

(2) Application stage of sport and health products.

**Figure 2:**
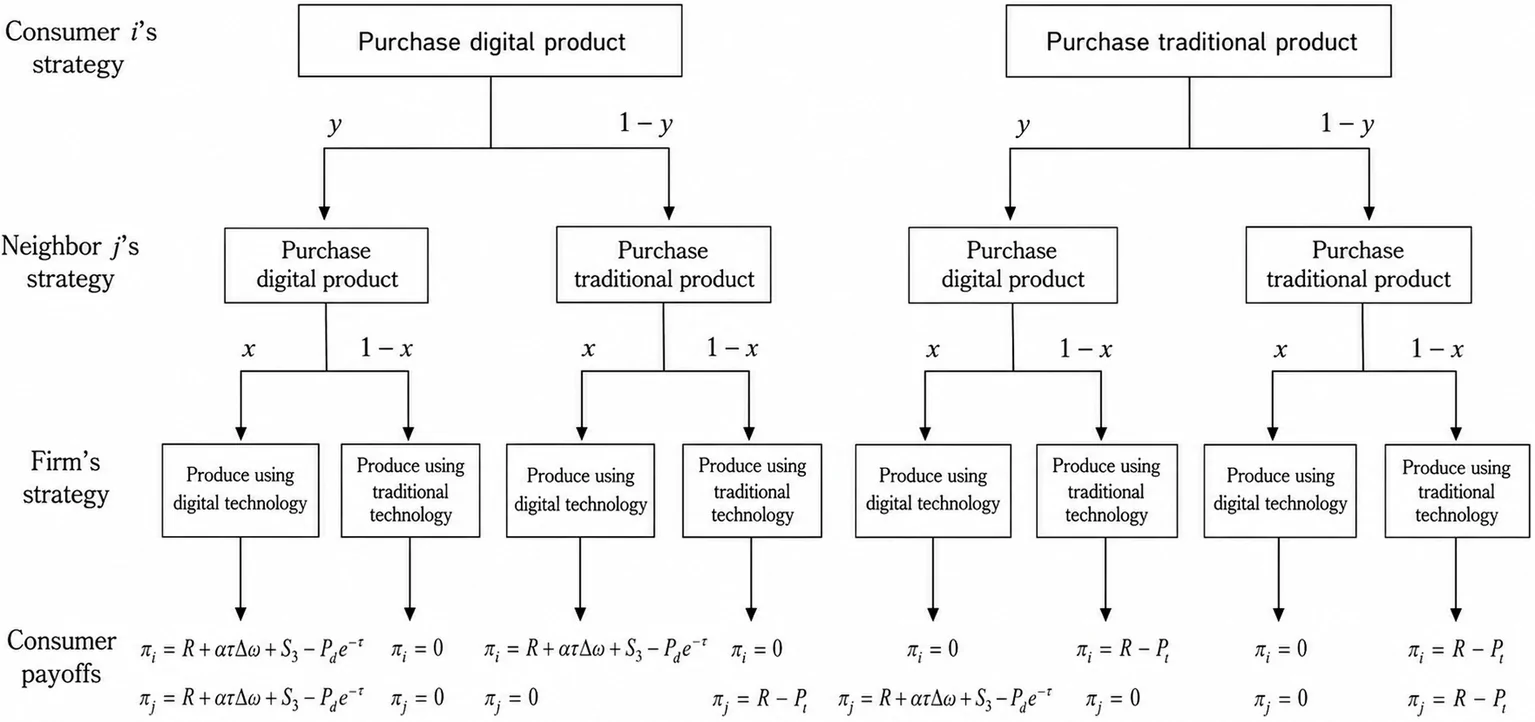
Strategy matrix of consumer adoption and firm response in the application stage of sport and health products.

Sports goods manufacturing firms promote the optimization and market entry of sport and health technologies, health monitoring devices, intelligent exercise equipment, and related products through collaborative innovation with universities and research institutions. After obtaining relevant information about sport and health products, consumers form an initial judgment about the extent to which these products can meet their personal needs based on technological maturity, health utility, and individual sport and health demands. On the basis of this judgment, consumers and their network neighbors may choose between two strategies: actively purchasing sport and health products or maintaining a rational wait and see attitude. In the figure, the first row represents the strategy choice of consumer individual i , while the second row represents the two alternative strategies of its directly connected network neighbor j . The third row reflects the strategy choices of leading firms and following firms between collaborative innovation and temporary non cooperation under the evolution of consumer behavior. The final row indicates the payoffs ultimately obtained by consumers during the interactive evolutionary process between consumers and firms (Figure 2).

### 3.3 Stability analysis of firms’ digital innovation in the sports goods manufacturing industry

#### 3.3.1 Structural configuration and parameter rationality

To examine the coevolution of digital transformation in the sports goods manufacturing industry and consumer adoption of digital sport and health products, a two-layer complex-network evolutionary game model was developed in MATLAB R2024a. To reduce stochastic effects arising from network generation and strategy updating, Monte Carlo simulations were repeated, and the average results were used for analysis. The upper layer represents a Barabási–Albert scale-free enterprise network, describing the evolution of digital technology adoption among leading and following firms, whereas the lower layer represents a Watts–Strogatz small-world consumer network, capturing product adoption through social learning and local interactions. This network structure reflects the heterogeneous characteristics of the enterprise system and the clustering and short-path diffusion patterns of consumer behavior.

Because the diffusion of digital sport and health products is influenced by multiple factors, some parameters cannot be directly obtained from a single data source. Therefore, parameter values were determined according to the payoff structure and economic interpretation of the model, normalized to ensure comparability between the enterprise and consumer layers, and subsequently calibrated to reproduce realistic diffusion patterns.

To better construct the model and reflect real-world conditions, this study determines the initial values accordingly. Observable parameters, such as product prices, production costs, and policy incentives, are mainly based on market reports and policy documents (34–49). Latent parameters, including digital orientation, consumer value perception, technological capability, innovation risk, cooperation costs, and competitive pressure, are assigned reasonable ranges based on existing literature (1, 4, 10–14, 18, 21, 26, 28–30, 33, 50–55), and the baseline values are ultimately determined through fitting and calibration. The final parameter settings are presented in Table 4.

**Table 4:** Parameter rationality and expected directional effects.

Parameter	Definition	Reasonable boundary	Initial value	Directional assumption	Expected effect on the model	Rationality explanation	Reference
*λ*	Enterprise preference for digital innovation	0 ≤ *λ*	0.5	*λ* ↑ → greater weight on digital returns	Promotes enterprise digital technology adoption and accelerates supply-side diffusion.	A stronger digital orientation increases the relative attractiveness of digital innovation and its long-term competitive benefits.	(1, 4, 10, 36, 37, 43)
*α*	Consumer preference for digital sport-health products	0 ≤ *α*	2.5	*α* ↑ → higher perceived digital incremental value	Raises consumer adoption probability, expands digital-product demand, and strengthens demand feedback to firms.	A stronger preference increases consumers’ valuation of monitoring, health feedback, and personalized services.	(21, 29, 30, 35, 40, 41, 43)
S₁	Subsidy for leading firms	S₁ ≥ 0	5	S₁ ↑ → higher net payoff from digital adoption	Promotes digital adoption among leading firms and strengthens early supply-side diffusion.	The subsidy reduces the investment burden and increases the net return from digital transformation.	(45, 53, 55)
S₂	Subsidy for following firms	S₂ ≥ 0	3	S₂ ↑ → higher net payoff from digital adoption	Promotes digital adoption among following firms and reduces the lag between leading and following firms.	The subsidy alleviates resource constraints and lowers the transition barrier faced by following firms.	(45, 46, 53, 55)
S₃	Consumer purchase subsidy	S₃ ≥ 0	2	S₃ ↑ → higher consumer adoption payoff	Raises resident adoption probability, enlarges market demand, and supports cross-layer diffusion.	The subsidy lowers the economic threshold for purchasing digital sport-health products and improves accessibility.	(42, 47, 53, 54)
Pd	Price of digital products	Pd > 0	13	Pd ↑ → lower digital-product demand	Suppresses consumer adoption and weakens the market incentive for enterprise digital adoption.	A higher purchase price reduces consumers’ willingness to adopt digital products.	(21, 29, 30, 35, 40, 41, 43)
Pt	Price of traditional products	Pt > 0	10	Pt ↑ → lower attractiveness of traditional products	Improves the relative competitiveness of digital products and indirectly promotes digital diffusion.	A rise in the traditional-product price narrows the price advantage of conventional products.	(18, 33, 36, 37, 43)
Cd	Cost of digital technology	Cd > 0	8	Cd ↑ → lower digital payoff for firms	Reduces enterprise digital adoption and slows supply-side diffusion.	Higher expenditure on R&D, intelligent production, and digital platforms lowers the net return from digital adoption.	(1, 10, 28, 36, 37, 40, 41, 45)
Ct	Cost of traditional technology	Ct > 0	5	Ct ↑ → lower payoff from the traditional strategy	Weakens the persistence of traditional production and indirectly promotes digital substitution.	Higher conventional production costs reduce the competitiveness of maintaining traditional production.	(1, 10, 36, 37)
*τ*	Digital technology maturity	0 ≤ *τ* ≤ 1	0.3	*τ* ↑ → lower effective cost and higher product value	Promotes firm adoption, raises consumer adoption, and accelerates coordinated diffusion across both layers.	Greater maturity improves technical reliability, production efficiency, and the service value of digital products.	(1, 10, 40, 41, 46, 51)
Δw	Incremental value generated by digital functions	Δw > 0	9	Δw ↑ → higher consumer adoption payoff	Raises consumer adoption probability, expands digital demand, and strengthens feedback to enterprise adoption.	Monitoring, health feedback, and personalized guidance create additional utility beyond basic sport-health value.	(13, 14, 30, 39–41)
R	Basic sport-health value	*R* > 0	6	R ↑ → higher overall product utility	Supports baseline consumer demand and prevents adoption from collapsing under weak digital preference.	This parameter captures the fundamental health utility of sport-health products independent of digital preference.	(13, 14, 38, 42)
*ŋ*_L_	Digital capability of leading firms	*ŋ*_L_ > 0	0.65	*ŋ*_L_ ↑ → lower effective innovation risk	Promotes digital adoption among leading firms and strengthens their diffusion-leading role.	Stronger technological and organizational capability improves risk absorption and implementation efficiency.	(4, 10, 36, 37, 40, 48, 51)
*ŋ*_F_	Digital capability of following firms	*ŋ_F_* > 0	0.5	*ŋ*_F_ ↑ → lower effective innovation risk	Promotes digital adoption among following firms and narrows the adoption gap between firm groups.	Capability improvement facilitates learning, technology absorption, and digital transformation among following firms.	(28, 45, 46, 48, 50, 52)
n	Innovation-risk cost coefficient	*n* > 0	1.5	n ↑ → lower digital payoff	Suppresses enterprise digital adoption and slows diffusion, especially for firms with weaker capabilities.	Higher uncertainty and risk-management expenditure reduce the net return from digital innovation.	(1, 28, 36, 37, 40, 41)
m	Inter-firm cooperation cost	*m* > 0	2.5	m ↑ → lower collaborative return	Weakens enterprise cooperation, slows technology spillovers, and reduces diffusion efficiency.	Coordination, knowledge sharing, and resource integration generate additional organizational costs.	(12, 45, 46, 50)
F	Competitive pressure on firms retaining traditional production	*F* > 0	4.5	F ↑ → lower payoff from the traditional strategy	Accelerates the shift from traditional production to digital adoption and promotes enterprise-side diffusion.	As digital adoption expands, traditional firms face stronger market displacement and competitive pressure.	(1, 10, 36, 37, 40, 43)
qd	Endogenous market demand for digital sport-health products	qd > 0	8	y(t), *α*, *τ*, and Δw ↑ → Qd(*t*) ↑; effective digital-product price ↑ → Qd(*t*) ↓	Higher digital demand increases firms’ expected returns from digital innovation and transmits consumer-adoption feedback to the enterprise layer.	As more consumers adopt digital sport-health products and perceive greater digital health value, firms anticipate a larger market for digital products.	(34, 35, 49)
qt	Endogenous market demand for traditional sport-health products	qt > 0	6	1 − y(*t*) ↑ → Qt(*t*) ↑; y(*t*) ↑ → relative traditional demand ↓	Higher traditional demand supports the payoff from traditional production, whereas declining traditional demand accelerates digital substitution.	As consumers shift toward digital products, the relative market demand for traditional products gradually declines.	(36, 37, 49)

The parameter settings satisfy the expected directional relationships among costs, prices, subsidies, technological capability, consumer preferences, and market demand, thereby ensuring the behavioral consistency and economic rationality of the evolutionary game model.

#### 3.3.2 Validation of the supply and demand feedback mechanism

**Figure 3:**
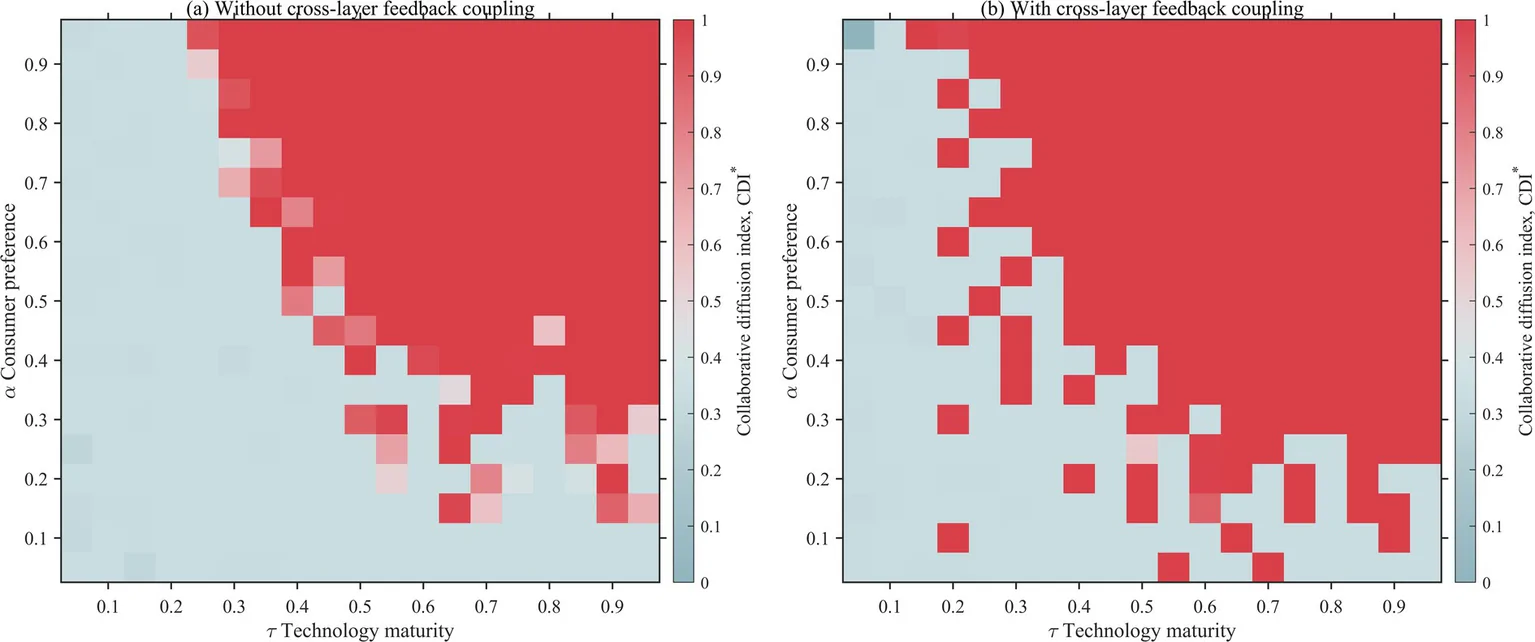
Validation of the supply and demand feedback mechanism.

To further examine the conditions under which the diffusion of digital sport and health products can be transformed into sustainable population health value, this study constructs two-dimensional parameter-scan heat maps with and without the supply–demand feedback mechanism. The horizontal axis represents the maturity of supply-side digital technology, whereas the vertical axis represents consumers’ value recognition of digital sport and health products. Color intensity indicates the final diffusion index. Rather than illustrating the linear effect of a single parameter, the heat maps identify the parameter regions under which digital innovation can achieve stable diffusion. The results show that incorporating the supply–demand feedback mechanism expands the high-diffusion region, indicating that continuous interaction between the supply and demand sides facilitates the transformation of digital product diffusion into sustainable population health outcomes. The results are presented in Figure 3.

#### 3.3.3 Validation using diffusion snapshots

**Figure 4:**
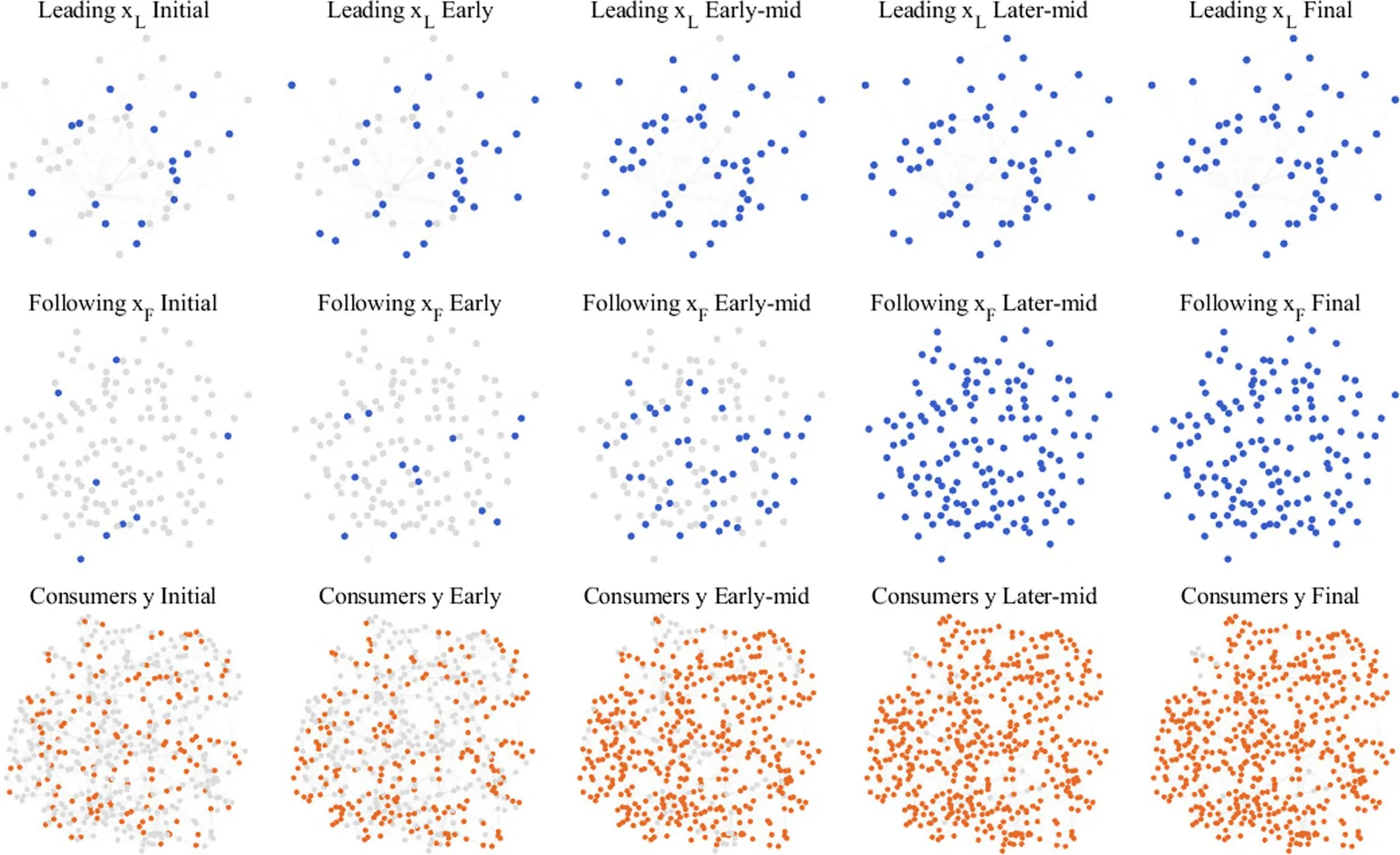
Diffusion snapshots of the two layer complex network.

To further validate whether the proposed two-layer complex-network model can reproduce a realistic diffusion process, snapshots of the strategy distributions in the enterprise and consumer networks were captured at five simulation stages (S1). As shown in Figure 4, strategy diffusion does not occur simultaneously across all nodes but instead evolves from local adoption to gradual network-wide diffusion. In the enterprise layer, leading firms adopt digital technologies earlier because of their stronger resource and innovation capabilities, subsequently driving following firms through demonstration and competitive effects. In the consumer layer, product adoption gradually expands through social interaction as the value of firms’ digital supply increases.

These diffusion snapshots demonstrate the dynamic rationality of the proposed model and illustrate the transmission pathway from firms’ digital technology adoption to consumer adoption. The results further suggest that sustained interaction between supply-side digital upgrading and demand-side adoption is essential for transforming localized diffusion into large-scale diffusion, thereby supporting broader access to sport and health services and promoting population health.

#### 3.3.4 Validation of evolutionary stability

Based on the replicator dynamic equations of the enterprise-side actors in (Equation 4-9), the partial derivatives are calculated and combined to construct the Jacobian matrix (J). By setting the replicator dynamic equations equal to zero, all equilibrium solutions of the system can be obtained. Specifically, by letting (*F*(*x*) = 0) and (*F*(*y*) = 0), five evolutionary equilibrium points of the decision-making dynamic system are derived, namely (0,0),(0,1), (1,0), (1,1), and the interior equilibrium point (*x*, *y*).


$$\begin{array}{c} J=[\begin{array}{cc} \frac{\partial F_{1}}{\partial \psi } & \frac{\partial F_{1}}{\partial \varphi }\\ \frac{\partial F_{2}}{\partial \psi } & \frac{\partial F_{2}}{\partial \varphi } \end{array}]=[\;\;\begin{array}{c} J_{1\;\;}\;J_{2}\;\\ J_{3}\;\;J_{4} \end{array}] \end{array}$$ (10)

According to the determinant and trace formulas, the determinant and trace of the Jacobian matrix (*J*) are calculated at the evolutionary equilibrium points shown in Equation (10). Based on the stability criteria (detj > 0) and (trj < 0), the evolutionarily stable strategy of the decision-making dynamic system can be identified (Table 5).

**Table 5:** Equilibrium points and stability analysis.

Eauilibrium points	detj	trj	Local stability results
(0,0)	−	−	Unstable point
(0,1)	+	+	Saddle point
(1,0)	+	+	Saddle point
(1,1)	+	−	Conditional ESS

**Figure 5:**
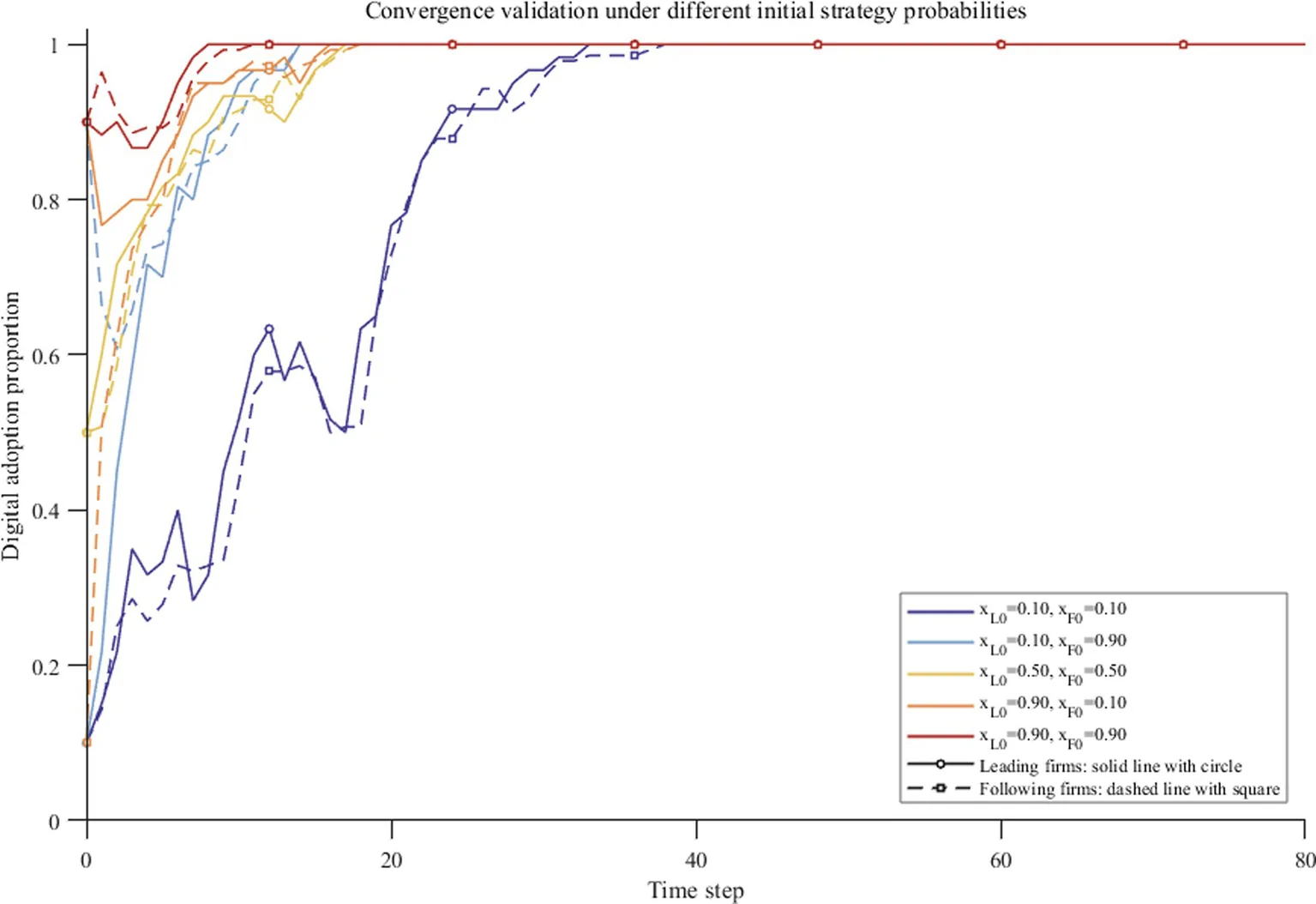
Comparison of simulated and observed consumer adoption.

The purpose of this study is to identify the optimal evolutionary state in which the transformation of sports goods manufacturing firms promotes the upgrading of sports goods and further facilitates sport and health consumption. The expected stable outcome is E (1,1). Based on realistic conditions, assumptions regarding variable relationships in previous studies, and relevant government policy documents, this study sets the value ranges of the parameters and conducts symbolic calculations of the eigenvalues. The signs of the eigenvalues are shown in Table 3, while the basic parameter values required for model validity are presented in Table 4. Therefore, the core conditions for the stable diffusion of digital innovation on the enterprise side are as follows: $\begin{array}{l} (p_{d}e^{-\tau }-c_{d}e^{-\tau })q_{d}+\lambda q_{d}+S_{1}+F+R+\text{ατΔw}>(p_{t}-c_{t})q_{t}+\frac{n\tau^{2}}{\eta_{L}}+me^{-x}\\ +R\;(p_{d}e^{-\tau }-c_{d}e^{-\tau })q_{d}+\lambda q_{d}+S_{2}+F+R+\text{ατΔw}>(p_{t}-c_{t})q_{t}+\frac{n\tau^{2}}{\eta_{F}}\\ +me^{-x}+R \end{array}$ . In summary, when consumers have strong digital health preferences, firms show a high preference for digital transformation, government subsidies remain moderate, technology maturity improves, and risk management costs decline, both leading firms and following firms tend to adopt digital innovation technologies. Under these conditions, the system eventually stabilizes at (1,1), indicating that the digital upgrading of the sports goods manufacturing industry achieves stable diffusion on the enterprise side. Conversely, when consumers’ digital health preferences are insufficient, firms’ preference for digital transformation is weak, subsidy incentives are inadequate, or the costs and risks of digital innovation remain high, firms are more likely to maintain traditional production technologies. In this case, the system may stabilize at (0,0), indicating an insufficient supply of digital sport and health products and a constrained diffusion of sport and health consumption. The evolutionary stability of the model is illustrated in Figure 5

#### 3.3.5 Empirical scenario validation

**Figure 6:**
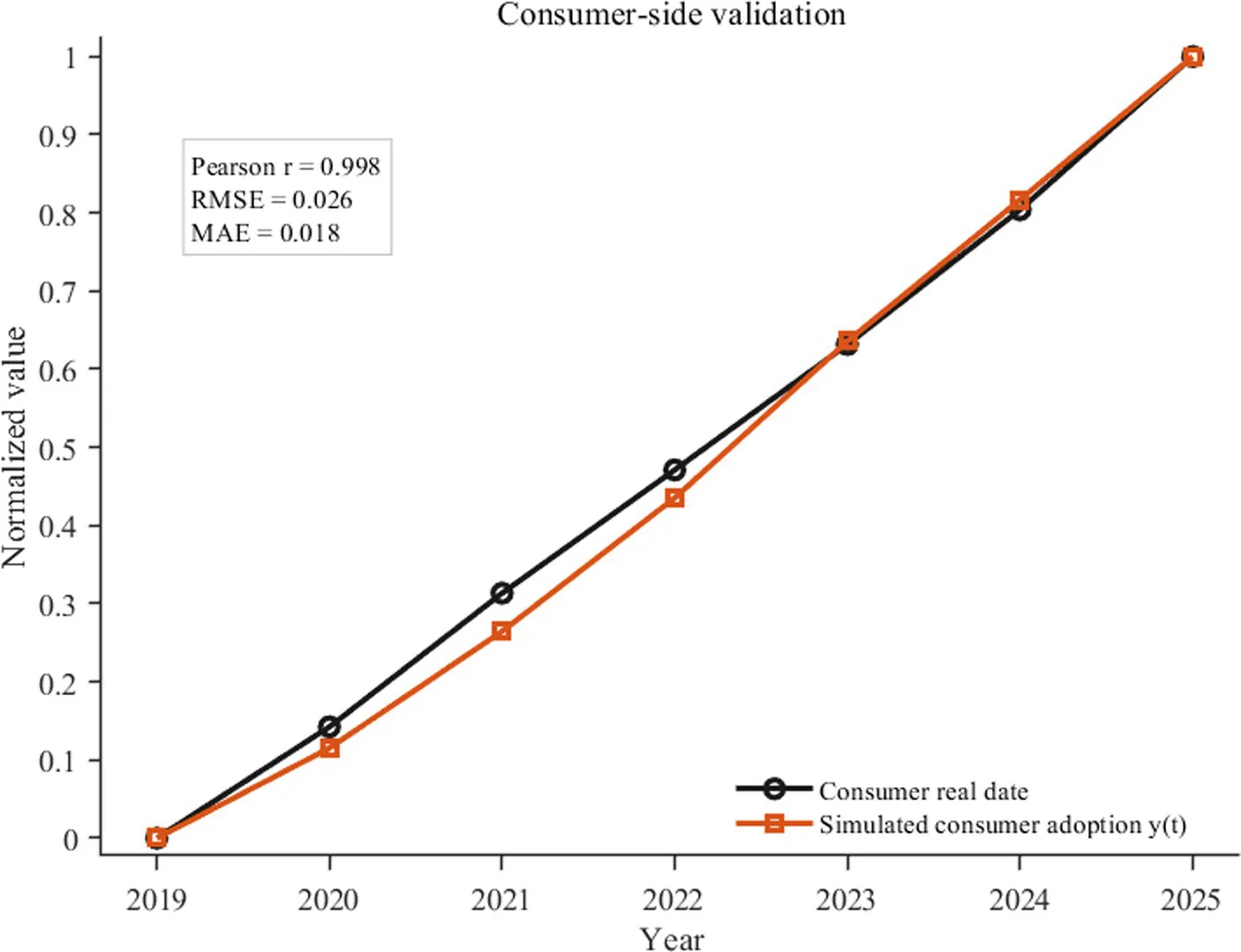
Comparison of simulated and observed digital technology adoption by leading firms.

**Figure 7:**
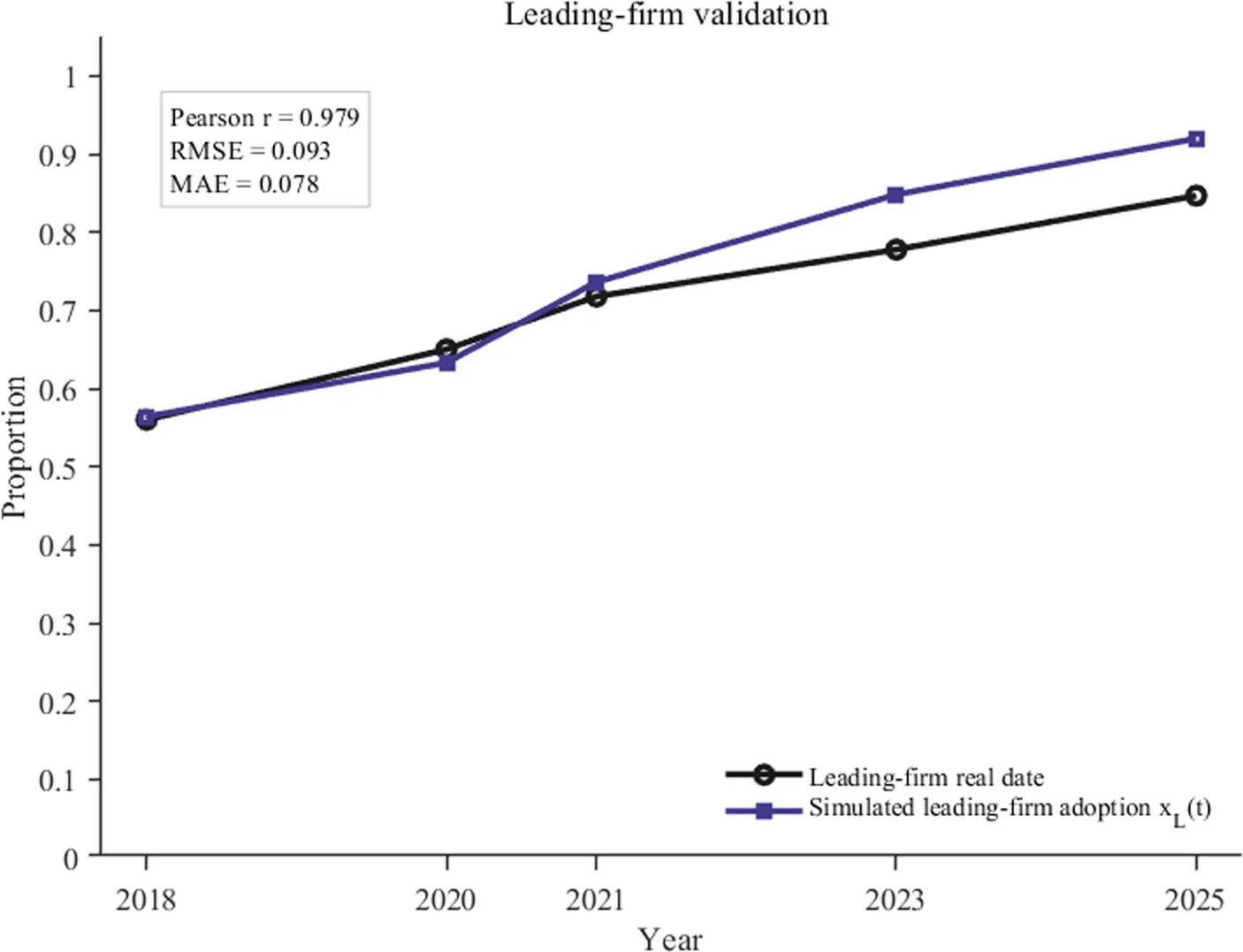
Comparison of simulated and observed digital technology adoption by following firms.

**Figure 8:**
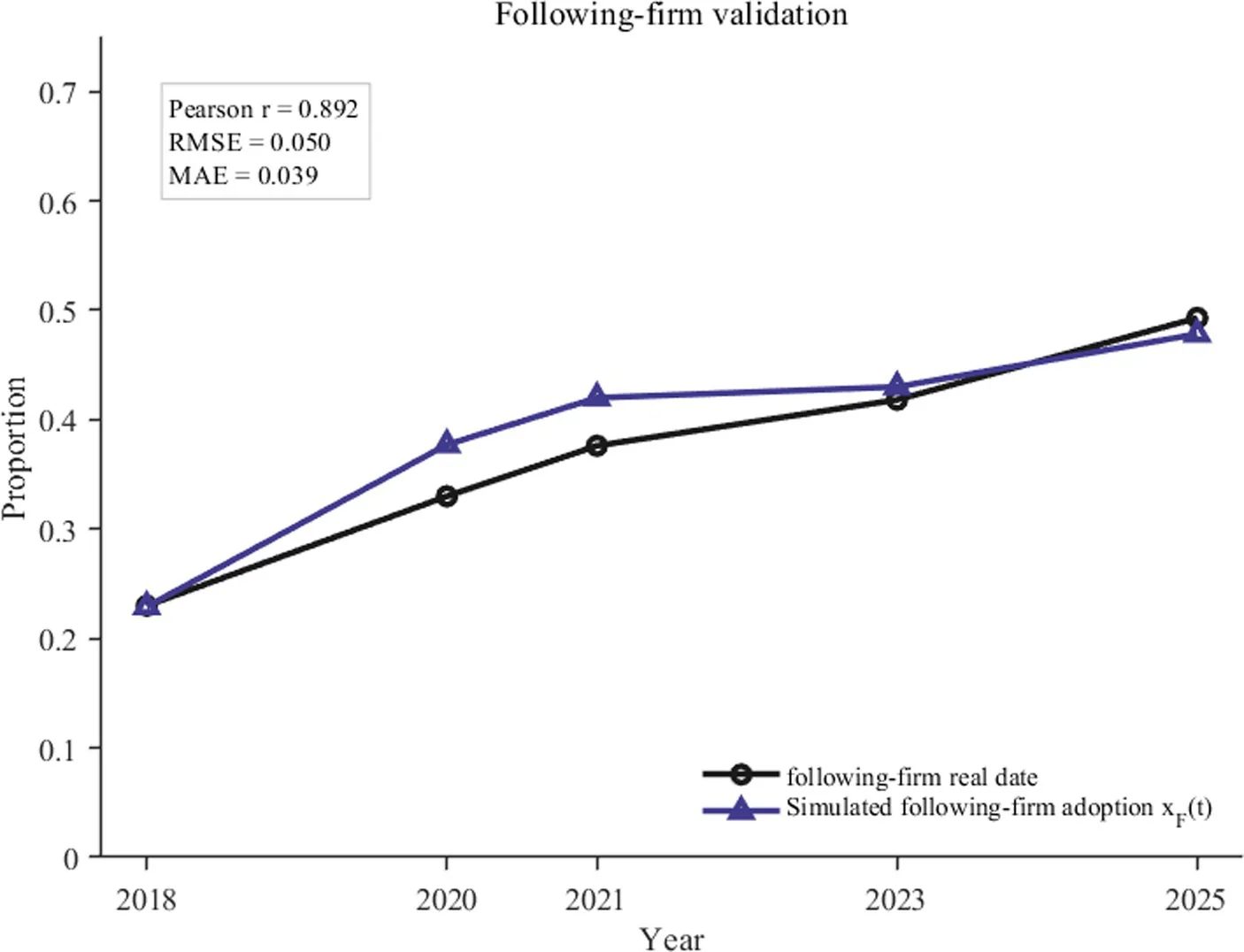
Evolution of the digital technology adoption rate among leading firms under policy incentives.

To further evaluate whether the proposed complex-network evolutionary model can reasonably capture the diffusion of digital sport and health products in both the supply and demand sides, external real-world data were introduced for scenario validation (Figures 6, 7, 8).

On the consumer side, global wearable device shipment data (2019–2025) released by IDC were used as a proxy for the diffusion of digital sport and health product adoption. Because wearable devices provide functions such as physical activity monitoring, health data recording, exercise feedback, and personalized health management, their market diffusion closely reflects consumers’ adoption of digital sport and health products. The fitting results showed high consistency between the simulation and real-world data, with a Pearson (0.998), an RMSE (0.026), and an MAE (0.018).

On the enterprise side, the proportions of enterprises purchasing cloud computing services reported by Eurostat for 2018, 2020, 2021, 2023, and 2025 were adopted as a proxy for digital technology adoption. Cloud computing represents a fundamental infrastructure supporting digital transformation, data analytics, and intelligent operation. Consistent with the model setting, enterprises were classified into leading and following firms according to enterprise size. The simulation results also exhibited good agreement with the empirical data, with Pearson (0.979 and 0.892), RMSE (0.093 and 0.050), and MAE (0.078 and 0.039) for leading and following firms, respectively (S1–4; Table 6). Overall, the validation results demonstrate that the proposed model can effectively reproduce the diffusion trends of digital sport and health products and the heterogeneous digital transformation processes of firms, thereby supporting the reliability of the model and providing a sound basis for the subsequent sensitivity analysis.

**Table 6:** Result.

Validation group	Model variable	Pearson *r*	RMSE	MAE
Consumer	y(t)	0.9984	0.0256	0.0184
Leading firms	xL(t)	0.9786	0.0927	0.0784
Following firms	xF(t)	0.8923	0.0500	0.0394

## 4 Results

### 4.1 Digital upgrading of sports goods manufacturing firms and the promotion of mass fitness consumption under policy incentives

Taken together, the three figures indicate that policy incentives jointly promote the diffusion of sport and health products from both the supply side and the demand side.

**Figure 9:**
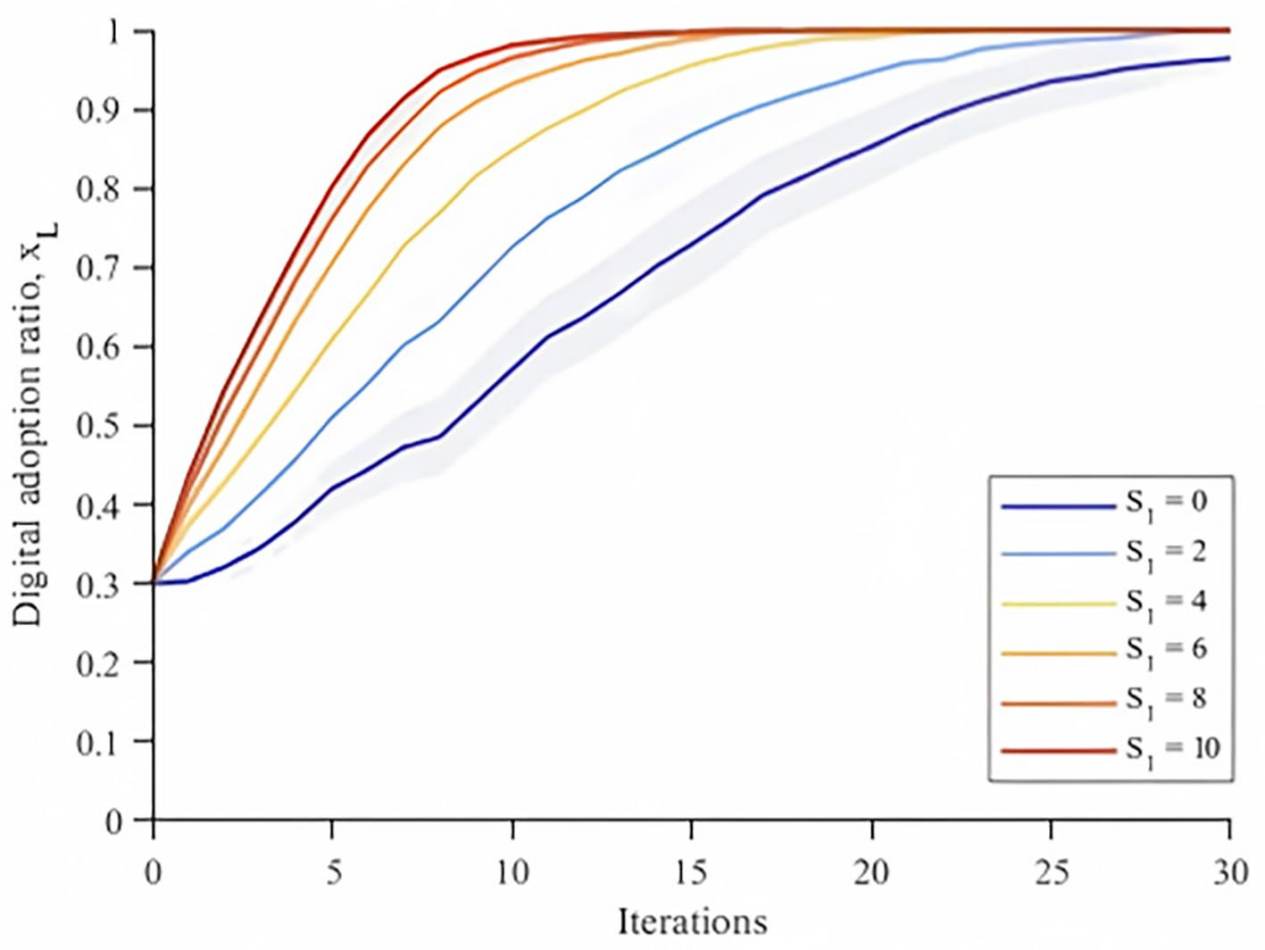
Evolution of the digital technology adoption rate among following firms under policy incentives.

The simulation results show that policy incentives have a positive effect on the digital transformation of both leading firms and following firms, although the two types of firms differ in response intensity and dynamic evolution. As shown in Figure 9a, when the level of enterprise subsidies is relatively low, leading firms still need to bear substantial technology research and development costs as well as innovation uncertainty. As a result, their technology adoption rate increases slowly during the early stage of diffusion, and the overall trajectory remains relatively flat, making it difficult to form a clear advantage in digital transformation. As subsidy intensity increases, especially after the subsidy level exceeds the critical incentive threshold for leading firms, the slope of the technology adoption curve rises markedly and rapidly converges toward a high level stable state.

Compared with leading firms, Figure 9b shows that following firms also respond positively to subsidy incentives, but their overall response is relatively weaker. This is mainly because following firms have comparatively limited technological reserves and resource endowments. Even under policy support, their technology adoption decisions remain constrained by capability limitations and risk expectations. Therefore, under a lower subsidy level, the adoption rate of following firms increases more slowly. As subsidy intensity rises, their adoption rate continues to increase, but the overall growth rate remains lower than that of leading firms, and the gaps among curves under different subsidy levels are more pronounced. This suggests that subsidy policies can effectively stimulate following firms to participate in digital diffusion. However, compared with leading firms, the marginal incentive effect for following firms shows a certain degree of lag, meaning that stronger policy support or a longer transmission period may be required before the effect becomes fully evident. At the same time, the evolutionary curves of both leading firms and following firms show a marginally diminishing pattern as incentive intensity increases. This indicates that a blind and continuous increase in subsidies may lead to declining policy efficiency. In practice, incentive policies should therefore be implemented in a differentiated and stage specific manner according to the development conditions of different types of firms.

**Figure 10:**
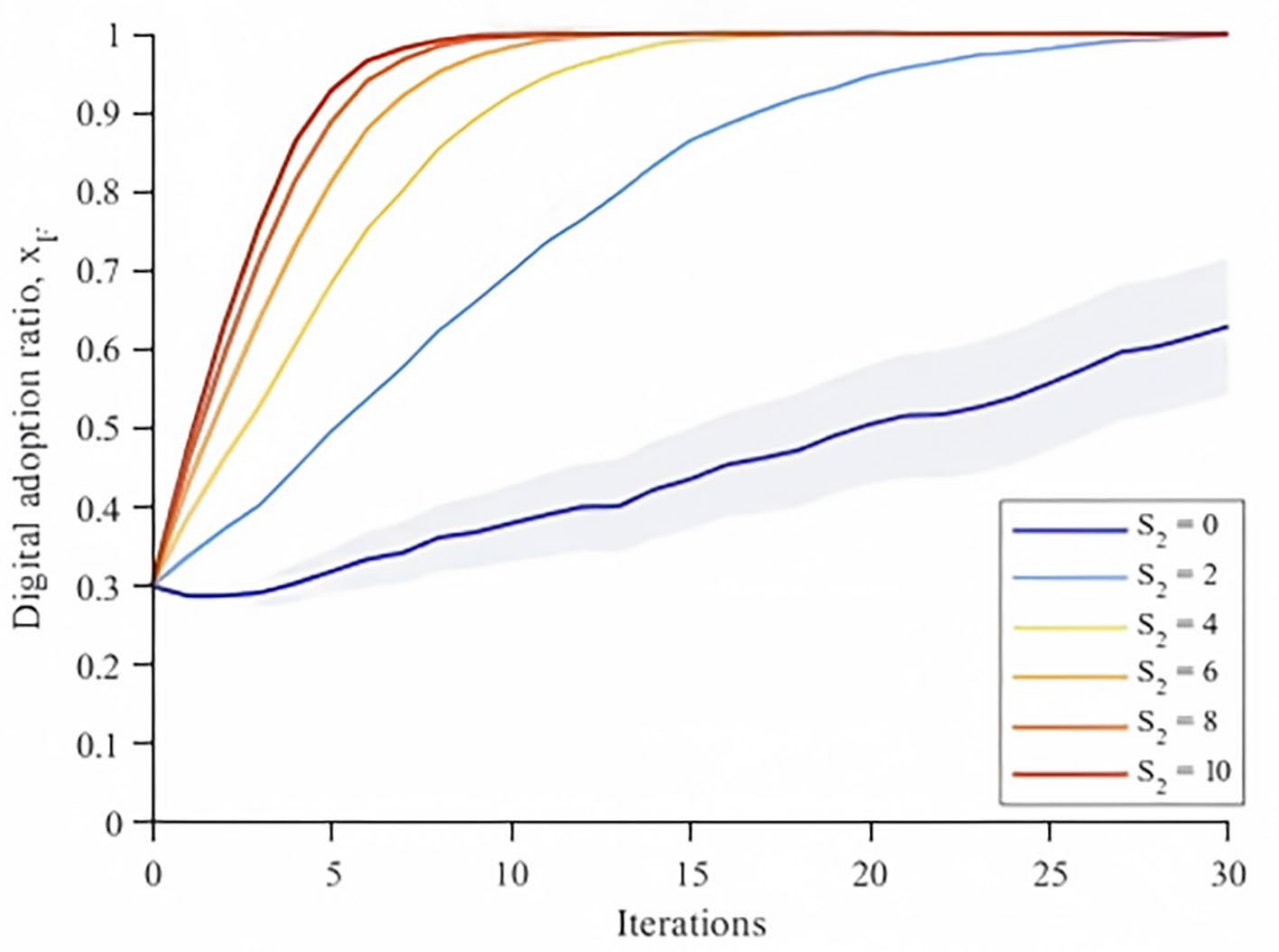
Evolution of the consumer adoption proportion of sport and health products under policy incentives.

From the consumer side, Figure 10 shows that consumer subsidies have a significant positive effect on the market diffusion of sport and health products. When the level of consumer voucher subsidies is low, consumers tend to have relatively weak purchase intentions because digital sport and health products remain relatively expensive and consumers still perceive certain usage risks and hold a wait and see attitude toward new products. Under this condition, the diffusion curve is relatively flat, and the proportion of consumer adoption remains at a low level for a long period. As subsidy intensity gradually increases, especially when S3 ranges from 2 to 4, the response intensity becomes the strongest. The actual purchase cost for consumers decreases substantially, and the perceived trial risk is effectively reduced, which further induces stronger purchase intention and a more pronounced diffusion effect. Accordingly, the curve shifts from slow growth to rapid increase and reaches a higher stable level within a shorter period. These results indicate that consumer side subsidies not only directly enhance consumers’ willingness to pay for sport and health products, but also accelerate the diffusion process and promote market penetration.

From the perspective of Innovation Diffusion Theory, the simulation results reflect the role of trialability in the innovation diffusion process. By reducing the economic costs and strategic risks associated with firms’ initial digital innovation and consumers’ product adoption, supply side and demand side policy incentives enable both firms and consumers to experiment with innovation under conditions of lower uncertainty. However, the diminishing marginal effects of policy incentives further indicate that enhanced trialability primarily facilitates the early stages of innovation diffusion, while in the long term, external incentives cannot substitute for the inherent relative advantage of the innovation itself.

### 4.2 Digital technology capability of firms and sport-health consumption

**Figure 11:**
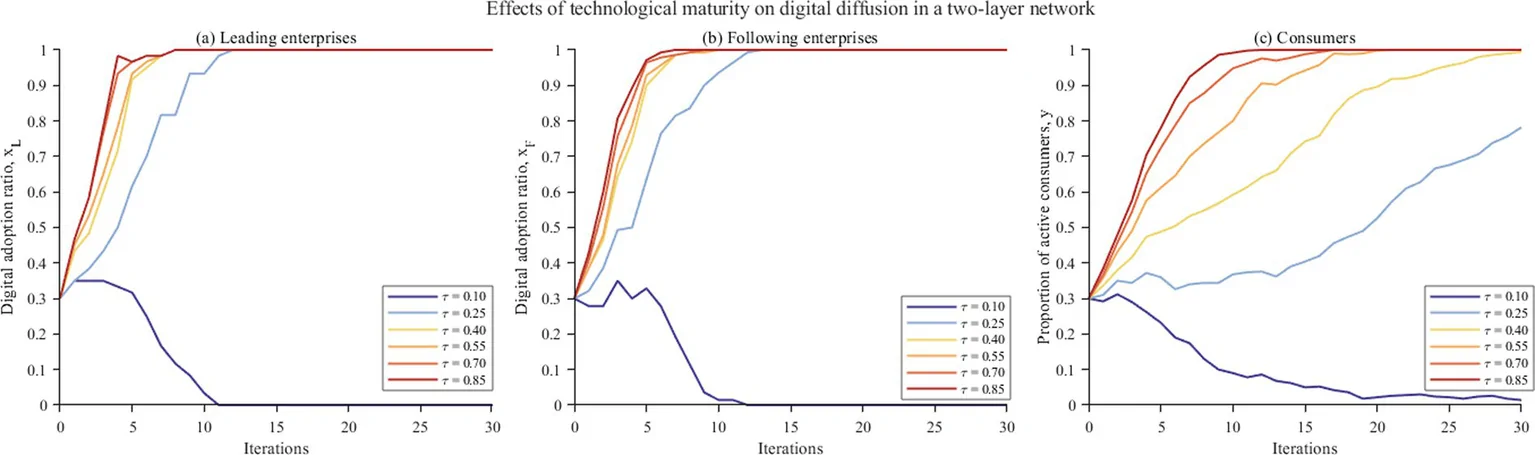
Evolutionary effects of technology maturity. **(a)** Digital technology adoption among leading firms **(b)** digital technology adoption among following firms **(c)** consumer adoption of sport and health products.

To examine the effect of firms’ digital technology capability on the diffusion of sport and health product consumption, this study sets the data analytics capability level of sports goods manufacturing firms as *τ* = (0.10, 0.25, 0.40, 0.55, 0.70, 0.85). A sensitivity analysis is conducted to investigate the evolutionary processes of firms’ digital technology adoption and residents’ product adoption under different levels of digital technology maturity. The results are shown in Figure 11.

As shown in Figures 11a,b, when *η* is at a relatively low level, the stability of digital products in research and development, health monitoring, and feedback functions remains insufficient. Under this condition, firms still need to bear relatively high research and development costs and innovation risks. At the same time, consumers may remain uncertain about the actual effectiveness of these products and find it difficult to obtain sufficient additional health utility. Therefore, under the low technology maturity scenario, the digital technology adoption rates of both leading firms and following firms increase slowly, while the proportion of residents actively purchasing sport and health products remains at a relatively low level for a long period. As technology maturity gradually improves, the operational stability of digital production and product functions is enhanced. Consequently, the costs of digital production and technology application decline, and the risk of innovation failure is also reduced.

At the firm level, higher technology maturity increases the relative benefits of adopting digital technology, making both leading firms and following firms more likely to choose digital technology strategies. Leading firms, supported by stronger technological foundations and resource integration capabilities, are usually able to achieve technological breakthroughs earlier and rapidly move toward a high level of adoption. Although following firms show a certain response lag, their digital technology adoption rate also increases gradually under the joint influence of technological cooperation and expanding market demand.

As shown in Figure 11c, at the resident level, the improvement of technology maturity not only enhances the user experience of digital sport and health products but also strengthens residents’ trust in intelligent recognition, health monitoring, and exercise feedback. The residents’ adoption curves show a relatively broad growth range, indicating that more mature technologies have a stronger attraction effect on residents. When *η* exceeds a certain threshold, the upward speed of the residents’ adoption curve increases markedly. This suggests that technology maturity may have entered a critical interval in which diffusion shifts from gradual improvement to accelerated expansion. At this stage, the health value provided by digital sport and health products can more effectively reduce residents’ perceived risks, thereby strengthening their purchase intention and further expanding market penetration through peer diffusion effects.

Greater technological maturity can simultaneously strengthen the relative advantage of an innovation and reduce its complexity. More mature digital technologies not only enhance consumers’ perceived functional value and health benefits but also reduce firms’ production costs, innovation risks, and technological uncertainty. Therefore, the accelerated diffusion observed once technological maturity exceeds a certain threshold suggests that innovation adoption may become self reinforcing when relative advantage continues to increase while perceived complexity declines to a sufficiently low level.

### 4.3 Effects of consumer preferences on sport-health consumption

**Figure 12:**
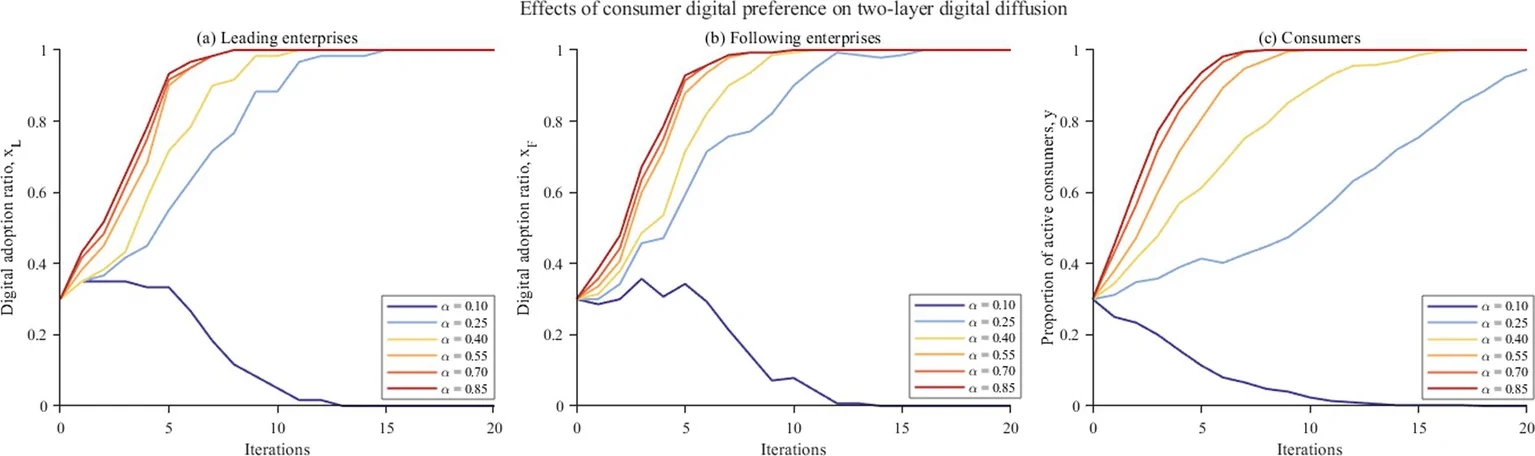
Evolutionary effects of consumer preferences: **(a)** digital technology adoption among leading firms; **(b)** digital technology adoption among following firms; **(c)** consumer adoption of sport and health products.

To examine the effect of changes in residents’ consumption preferences on the diffusion of digital sport and health products, this study sets the preference intensity of residents for digital sport and health products as *α* and analyzes the evolutionary characteristics of firms’ digital technology adoption rates and residents’ adoption proportion of sport and health products. The results are shown in Figure 12.

Figure 12c shows that when *α* is at a relatively low level, residents pay limited attention to personalized exercise functions such as intelligent physical activity monitoring. They are more likely to maintain existing consumption habits or adopt a rational wait and see strategy, resulting in a diffusion curve that remains at a relatively low level. As *α* gradually increases, residents attach greater importance to digital functions and their health promoting value. The expected utility of the product gradually exceeds the perceived risk costs, and the diffusion curves begin to diverge clearly and rise at an accelerated rate. In addition, digital sport and health products have strong experience good attributes. Residents’ purchase decisions depend not only on their own judgment of product functions and health value, but also on the usage experiences and evaluations of surrounding consumers. Through this process, individual purchase decisions are gradually transformed into group level adoption diffusion.

The strengthening of residents’ preferences affects not only the consumer side but also firms’ digital technology adoption decisions through market demand feedback. Figure 12a shows that when residents’ preference intensity is low, the growth of the digital technology adoption rate among leading firms is relatively limited. Although leading firms have stronger technological foundations and resource integration capabilities, weak resident demand indicates an insufficient potential market for digital sport and health products, which reduces firms’ motivation to invest in digital transformation. As *α* increases, market demand expands accordingly, and the expected returns from adopting digital technology increase significantly for leading firms. Their adoption curves therefore shift upward rapidly and show a faster convergence pattern. Figure 12b further shows that, under the low preference scenario, following firms are more likely to adopt a cautious strategy because of technological and resource constraints, and their adoption rates remain at a low level for a relatively long period. As residents’ preferences gradually increase, following firms improve their digital technology adoption rates by observing and learning from the digital transformation outcomes of leading firms and by relying on technological and industrial collaboration. However, compared with leading firms, following firms still need a longer process of technological learning, resource accumulation, and strategic adjustment. As a result, their curve responses show a certain degree of lag.

Overall, residents’ preferences are not merely isolated consumer psychological factors. They serve as an important link between residents’ health demands and firms’ digital supply. Only when residents develop a higher level of value recognition and willingness to use digital sport and health products can a more stable two way mechanism be formed between firms’ digital technology adoption and the consumer side diffusion of health oriented products.

The present evolutionary results indicate an interaction between compatibility and observability. As positive product adoption experiences become observable to potential consumers through consumer networks, they continuously update their perceptions of product value, thereby further reinforcing the diffusion process. The resulting expansion of market demand, in turn, feeds back into firms’ expected returns from innovation, ultimately forming a bidirectional feedback mechanism between the supply and demand sides.

## 5 Discussion

### 5.1 Main findings

Using a two-layer complex-network evolutionary game model involving firms and consumers, this study analyzed the dynamic relationship between digital innovation among sports goods manufacturing firms and residents’ adoption of sport and health products. The findings reveal not merely the isolated effects of policy incentives, technological capability, and consumer preferences on the speed of product diffusion, but also the process through which supply actors and users jointly shape the health value of digital technologies after they enter the field of population health under specific social and institutional conditions. Accordingly, this study argues that the diffusion of digital sport and health products is not simply a process of market penetration for ordinary consumer goods. Rather, it is a social process through which technological resources are transformed into population health capabilities.

Digital innovation by firms changes the ways in which residents access sport and health information and lifestyle services, while consumers reshape the direction of firm development through demand feedback and social diffusion. Therefore, the diffusion of digital sport and health products involves a continuous two way feedback process between supply and adoption.

Unlike studies that focus only on the market penetration rate of digital products, this study uses a dynamic two-layer network framework to examine how digital sport and health products may be transformed into supportive resources for public health. Their public health value does not depend solely on the number of products sold. More importantly, it depends on whether these products are aligned with population health needs, whether they can be accessed fairly by different social groups, and whether residents can use them continuously and effectively in real life settings.

### 5.2 Theoretical contributions

In the process of public health promotion, firms’ digital innovation can improve product quality, promote the broad adoption of sport and health products, and further influence physical activity levels and health outcomes through mechanisms such as health management. Product diffusion is a key part of this process. However, market diffusion can be translated into population health benefits only when products are used continuously and genuinely transformed into health related behaviors.

The analysis of policy incentives shows that external incentives act on both the supply side and the demand side of digital health products. A representative example is the WHO Global Strategy on Digital Health (2020–2025), which emphasizes that policy support should simultaneously strengthen digital health infrastructure, improve public accessibility, and promote equitable use of digital technologies. This supports our finding that coordinated incentives targeting both the supply and demand sides are more effective than single-sided interventions. However, different levels of incentive intensity and different stages of implementation may lead to different evolutionary outcomes. Single sided incentives are often insufficient to generate stable diffusion effects (53, 54). If subsidies are provided only to firms, they may reduce the cost of digital innovation and promote the supply of digital products to some extent. However, if product usability and public trust are not improved at the same time, digital products may become disconnected from residents’ actual health needs (55). Conversely, if subsidies are provided only to consumers, they may stimulate purchasing behavior in the short term. Yet, if the products themselves do not fit the diverse health needs of different populations, such consumption may remain a one time purchase and may not be converted into sustained population health benefits.

Technological maturity, on the one hand, strengthens relative advantage by enhancing the health benefits and service quality of products; on the other hand, it reduces perceived complexity by lowering technological uncertainty and difficulties in product use. Furthermore, improvements in digital technology maturity can enhance the precision and adaptability of exercise based health promotion. The practical value of technological maturity has also been demonstrated in real-world digital health applications. The Apple Heart Study provided evidence that wearable technologies equipped with advanced sensing and data analytics capabilities can support continuous cardiovascular monitoring and personalized health management at the population level. This example indicates that technological progress enhances not only product performance but also the capability of digital sport and health products to generate sustained public health value, which is highly consistent with the findings of this study. People of different ages and health conditions have heterogeneous exercise needs. Digital sport and health products can provide more targeted exercise recommendations through continuous analysis of physical activity behavior and health information, thereby helping such products become more integrated into residents’ everyday life settings (56, 57). However, stronger digital capability does not necessarily produce equal health benefits. People who use digital devices more frequently are more likely to be identified by algorithms and to receive personalized services. By contrast, groups with limited digital skills often lack complete data records and may therefore be marginalized within digital service systems (3, 58). At the same time, data related to physical indicators and lifestyle habits are highly private. Expanding the scope of data collection may also increase risks such as information leakage. Therefore, firms’ digital transformation must proceed together with public data governance (59). Firms need not only to improve data analytics capabilities but also to adhere to the principle of fairness and actively cover older adults and other vulnerable groups (60). Governments and industry organizations should establish data security regulatory standards for digital sport and health products to prevent technological precision from being achieved at the expense of health equity and individual rights. Only when responsibility protection and equity oriented governance are improved together can firms’ data analytics capabilities be truly transformed into public health value.

Consumer preferences should not be understood merely as fixed individual tendencies, but rather as consumers’ dynamic evaluations of an innovation’s relative advantage and compatibility. Consumers are more likely to adopt digital sport and health products when the expected health benefits and service value outweigh the perceived economic costs, usage costs, and privacy risks, and when the product functions align with their existing health needs, value perceptions, and physical activity habits (61). Residents’ attitudes toward digital sport and health products can significantly influence their dissemination and adoption among the population. Therefore, consumer preference should not be regarded as a fixed individual attribute.

First, health literacy determines whether residents can truly understand the health recommendations provided by these products (62). Even if a product has rich sport and health monitoring functions, its health impact will remain limited if residents cannot understand the indicators or translate data feedback into concrete actions (63). Second, trust is a key condition for the sustained adoption of digital health products (22). Residents need to trust not only the accuracy of product measurements and exercise recommendations, but also the way firms handle personal health data. Once there is a gap between product claims and actual effects, or once data use becomes irregular, residents’ willingness to use the product may decline rapidly (64). Third, consumer preferences are continuously shaped by social life. For example, the global success of Peloton demonstrates that consumers’ preferences for digital sport and health products are not determined solely by their initial purchase intentions, but are continuously shaped and reinforced through ongoing social interaction and shared experiences. As users share their exercise achievements, usage experiences, and product evaluations, potential consumers continuously update their perceptions of the product’s value and actual health benefits, thereby further strengthening their willingness to adopt digital sport and health products. Word of mouth and evaluations from surrounding users can constantly change an individual’s perception of a product (65). When products are supported in family, workplace, or exercise settings, residents are more likely to develop long term use habits. By contrast, when shared participation environments are lacking, residents may discontinue use even after completing an initial purchase. Therefore, improving consumer preferences requires coordinated efforts in health literacy, trust building, and integration into everyday life settings. Firms should translate professional health data into clear behavioral recommendations and communicate product functions in an accurate and transparent manner (66). Public health departments and community organizations can help residents understand and use relevant tools through digital health education and community based exercise programs. For older adults and groups with weaker digital capabilities, offline guidance, simplified interfaces, and supportive services should also be provided. Ultimately, the key to promoting the diffusion of digital sport and health products lies not only in making residents interested in technology itself, but also in ensuring that products respond to residents’ real health needs.

On this basis, policy design should shift toward more practical policy packages and should be dynamically adjusted according to the stage of industrial development. In the early stage of diffusion, policy efforts should focus on reducing technological uncertainty and initial adoption costs, thereby creating opportunities for firms and consumers to experiment with and experience digital innovations. In the middle stage, policy priorities should shift toward improving product compatibility and enhancing the health benefits generated by adoption. In the later stage, greater attention should be given to sustained use and equitable population coverage. Accordingly, policy objectives should gradually move beyond market expansion toward improving the accessibility of health support resources and generating broader population health benefits. Public health departments should not stop at external regulation of products, but should participate more deeply in the evaluation of health effects (67, 68). Firms should assume responsibility for product quality and data security, while communities and schools should serve as intermediary organizations that help products enter real health settings (69).

### 5.3 Public health implications: from digital product diffusion to the formation of population health capabilities

Improvement in any single factor is insufficient to guarantee the realization of public health value. Only when the supply side can provide reliable and adaptive products, the demand side can access and correctly use these products, and the institutional environment can safeguard fairness and trust, can the market diffusion of digital products be further transformed into population health benefits. From this perspective, the adoption of digital sport and health products is not merely a market purchasing behavior. It is a process of health capability formation, through which residents use digital tools to internalize external technological resources into practical abilities to perceive their own health status, make health decisions, and maintain health behaviors.

This study argues that the diffusion of digital sport and health products should be understood as an important part of the historical development of population health, rather than as a simple commercial scenario. Historically, exercise based health promotion has long relied on place based interventions, such as public sport facilities, community activities, school physical education, and medical advice. Today, the development of digital technology allows health support to extend into the everyday life settings of the public and transforms health feedback into a more continuous form of companionship.

Overall, the real value of digital sport and health products does not lie in creating more products for sale. Rather, it lies in whether these products can genuinely change the ways in which residents take health related actions. Only when digital technology is transformed from a commercial technological resource into residents’ real health action capability, while balancing efficiency, trust, and equity in this process, can it contribute to the realization of population health.

## 6 Conclusion

(1) Policy incentives can effectively promote technological transformation among sports goods manufacturing firms and activate the sport and health consumption market. However, government subsidies on the firm side show diminishing marginal effects, suggesting that a reasonable subsidy threshold should be established.
(2) High-quality digital sport and health products are a core factor driving the diffusion of sport and health consumption. Improvements in firms’ digital technology capability and product technology maturity can enhance health utility, user experience, and market attractiveness.
(3) When residents develop strong value recognition and willingness to use digital sport and health products, a more stable mutually reinforcing mechanism can be formed between firms’ digital technology adoption and consumer-side diffusion of health-oriented products. Conversely, under low-preference conditions, both the supply side and the demand side are likely to remain trapped in a low-level diffusion state.

### 6.1 Limitations and future research

Although this study provides an analytical framework for understanding the coevolutionary relationship between the digital upgrading of the sports goods manufacturing industry and the diffusion of sport and health consumption, several limitations should be acknowledged. The model mainly captures the evolutionary relationship between firms’ strategic choices and consumers’ product adoption, but it does not directly incorporate sustained use behavior, changes in physical activity, or actual health outcomes. Therefore, this study offers a mechanistic explanation of how digital sport and health products may be transformed into public health value, rather than direct evidence of improved health outcomes. The parameter settings were designed to ensure that the model could reasonably capture the evolutionary dynamics and diffusion trends of digital sport and health products. Future research could further refine the model using richer empirical data. Future research could further incorporate consumer heterogeneity, including digital literacy, socioeconomic status, age, urban–rural differences, and health risks, to examine how different population groups vary in the transformation of digital health value. Longitudinal empirical studies are also needed to evaluate the long-term effects of digital sport and health products on physical activity levels and health outcomes.

## Data Availability

The original contributions presented in the study are included in the article/supplementary material, further inquiries can be directed to the corresponding authors.
